# Effect of temperature change on the performance of the hybrid linear flow channel reactor and its implications on sulphate-reducing and sulphide-oxidising microbial community dynamics

**DOI:** 10.3389/fbioe.2022.908463

**Published:** 2022-08-26

**Authors:** T. S. Marais, R. J. Huddy, R. P. Van Hille, S. T. L. Harrison

**Affiliations:** ^1^ Department of Chemical Engineering, Centre for Bioprocess Engineering Research, University of Cape Town, Cape Town, South Africa; ^2^ The Future Water Institute, University of Cape Town, Cape Town, South Africa; ^3^ The Moss Group, Cape Town, South Africa

**Keywords:** biological sulphate reduction, partial sulphide oxidation, microbial community succession, 16S rRNA amplicon sequencing, floating sulphur biofilm

## Abstract

Semi-passive bioremediation is a promising strategy to mitigate persistent low volume mine-impacted wastewater containing high sulphate concentrations. Building on the proof of concept demonstration of the hybrid linear flow channel reactor (LFCR), capable of simultaneous biological sulphate reduction and partial sulphide oxidation with elemental sulphur recovery, the impact of key operating parameters, such as temperature, on process performance is critical to real-world application. Temperature fluctuates seasonally and across the diurnal cycle, impacting biological sulphate reduction (BSR) and partial sulphide oxidation. The process is reliant on the metabolic activity and synergistic interactions between sulphate-reducing (SRB) and sulphide-oxidising (SOB) microbial communities that develop within discrete oxic and anoxic microenvironments within the hybrid LFCR. In this study, the impact of operating temperature on process performance was evaluated by decreasing temperature with time from 30 to 10°C in each of three laboratory-scaled hybrid LFCR units operating in pseudo-steady state at 1 g/L sulphate. Using lactate as a carbon source, two reactor sizes (2 and 8 L) were considered, while the impact of lactate vs. acetate as carbon source was evaluated in the 2 L reactors. On incremental decrease in temperature from 30 to 10°C, a decrease in volumetric sulphate reduction rate was observed: from 0.144 to 0.059 mmol/L.h in the 2 L lactate-fed reactor; from 0.128 to 0.042 mmol/L.h in the 8 L lactate-fed reactor; and from 0.127 to 0.010 mmol/L.h in the 2 L acetate-fed reactor. Similarly, sulphate conversion efficiency decreased (2 L lactate-fed: 66% to 27%; 8 L lactate-fed: 61% to 20%; 2 L acetate-fed: 61% to 5%). A decrease in temperature below the critical value (15°C) led to considerable loss in metabolic activity and overall BSR performance. Sessile and planktonic microbial communities were represented by bacterial phyla including Proteobacteria, Synergistetes, Bacteroidetes, and Firmicutes. A diverse group of putative SRB (Deltaproteobacteria) and SOB, including Alpha, Beta, Gamma, and Epsilonproteobacteria phylotypes, were prevalent and shifted in relative abundance and community composition in response to decreasing temperature. Specifically, the decrease in the relative abundance of Deltaproteobacteria with decreasing temperature below 15°C corresponded with a loss of BSR performance across all three reactors. This study demonstrated the impact of low temperature on the physiological selection and ecological differentiation of SRB and SOB communities within the hybrid LFCR and its implications for real-world process performance.

## 1 Introduction

The application of semi-passive biochemical reactors is a promising strategy for mitigating sulphate-rich effluents (Sánchez-Andrea et al., 2014). These systems are ideal for treatment of persistent, low volume discharge in remote locations where limited infrastructure and expertise are available (McCarthy, 2011; Pat-Espadas et al., 2018). However, the widespread application of these processes is often limited due to challenges such as reaction kinetics, sulphide management and the provision of a cost-effective carbon substrate (Harrison et al., 2014; Yang et al., 2021).

The development of an integrated semi-passive process, based on a single-stage hybrid linear flow channel reactor (LFCR), capable of simultaneous biological sulphate reduction and partial oxidation of sulphide to elemental sulphur is a promising approach to overcoming the limitations of current treatment strategies (Marais et al., 2020a). The process relies on the formation of niche microenvironments comprising an microaerobic zone at the air-liquid interface and an anoxic zone within the bulk volume of the LFCR. Under ideal conditions the synergistic interaction between sulphate-reducing (SRB) and sulphide-oxidising bacterial (SOB) consortia can facilitate a linear biotransformation of sulphate to elemental sulphur (Marais et al., 2020b). In the hybrid LFCR, SRB in the anoxic bulk volume reduce sulphate in the presence of a suitable carbon substrate and electron donor to hydrogen sulphide (HS^−^). The lack of turbulent mixing within the bulk volume results in the formation of a sulphide gradient, while the floating biofilm impedes oxygen mass transfer into the reactor, creating an ideal pH and redox environment to support the biologically catalysed partial oxidation of sulphide to elemental sulphur within the biofilm (Mooruth, 2013). Biofilm formation is initiated by the colonisation of the air-liquid interface by heterotrophic bacteria that produce extracellular polymeric substances (EPS) followed by the incorporation of an active SOB population, resulting in the formation of a floating sulphur biofilm (FSB). The co-existence of SRB and SOB communities are well documented, particularly in environments such a marine sediment (Zhang et al., 2017), petroleum reservoirs (Tian et al., 2017), and sewerage systems (Dong et al., 2017).

Previous investigations have reported on the proof-of-concept, development and characterisation of the hybrid LFCR process under different operating parameters, such as the effect of hydraulic residence time (HRT), carbon substrate and reactor geometry (Marais et al., 2020a; Marais et al., 2020b; Marais et al., 2020c). These studies have highlighted the potential of the hybrid LFCR as an alternative semi-passive technology, achieving relatively high sulphate and sulphide removal efficiencies with the recovery of elemental sulphur as a value-adding by-product. The initial development and characterisation of the hybrid LFCR was performed under temperature controlled conditions. An important consideration for real-world implementation is the inability to control temperature, making the system susceptible to diurnal and seasonal fluctuations. Therefore, the performance of the hybrid LFCR process under varying temperature regimes, typical for temperate climate conditions, needs to be assessed to determine its feasibility for large-scale application.

The effect of temperature on sulphate reduction and sulphide oxidation have been well described in literature (Moosa et al., 2005; Naegele et al., 2013; ben Ali et al., 2020). Most studies evaluated BSR and sulphide oxidation within separate reactor systems, including continuously stirred-tank reactors (CSTR) (Moosa et al., 2005; Bijmans et al., 2008), fixed bed trickling reactors (FTBR) (Naegele et al., 2013), an anaerobic side-stream reactor (Ferrentino et al., 2017), and expanded granular sludge bed reactors (EGSB) (Sposob et al., 2017). However, the effects of temperature on the hybrid LFCR process incorporating both BSR and sulphide oxidation has not been reported. Microbial activity in response to temperature is dictated by upper and lower limits of temperature for growth (Ferrentino et al., 2017). Most SRB and SOB have been characterised as mesophiles, with an active temperature range typically between 10 and 40°C and an optimum temperature around 30°C (Greben et al., 2002). Higher operational temperatures increase metabolic reaction rates and overall system performance, provided the temperature remains within the optimal range for both SRB and SOB activity. A decrease in temperature results in reduced overall kinetic performance up to a critical point, below which further prolonged decrease in temperature would result in significant loss in microbial activity and performance, eventually leading to system failure.

The influence of changes in microbial community structure is an essential component to understanding the biocatalytic sulphur transformations occurring within the hybrid LFCR. The hybrid LFCR facilitates discrete microenvironments that develop diverse sessile and planktonic microbial populations, localised across different zones of the reactor (Marais et al., under review). Key factors that have been shown to influence the microbial community composition within the hybrid LFCR are associated with steep chemical gradients (oxygen and sulphide), hydraulic residence time, carbon substrate and physiological state (planktonic and sessile) of the bacteria (Marais et al., under review). Similarly, due to the impact of temperature on microbial metabolism, the response of the microbial community to changes in environmental temperature is particularly important to gain new insight into the spatial and temporal shift in SRB and SOB communities within the hybrid LFCR. This will help to evaluate the extent to which observed changes in performance are due to reduced metabolic rate, changes in community structure, or both. The information is important in predicting the effects of seasonal and diurnal temperature on process performance and robustness.

In this paper, we investigate the range of temperatures likely to be experienced in a typical passive wastewater treatment process in a seasonally temperate environment in terms of sulphate reduction and partial sulphate oxidation performance in the LFCR. The research extends the characterisation and understanding of the SRB and SOB relationship within the hybrid LFCR. In addition, the impact of reactor geometry and carbon source are evaluated. These findings are key for the design of the scaled-up reactor.

## 2 Materials and methods

### 2.1 Microbial cultures

The mixed SRB consortia has been maintained at the University of Cape Town (UCT) on sterile, modified Postgate B (MPB) medium (0.46 g/L KH_2_PO_4_; 1.0 g/L NH_4_Cl; 1 g/L MgSO_4_.7H_2_O; 0.9 NaSO_4_; 0.4 g/L yeast extract; 0.3 g/L sodium citrate (Oyekola et al., 2012). The carbon source/electron donor was supplemented at a 0.7 COD/SO_4_^2-^ ratio with either 50% (w/w) sodium lactate (1.92 ml/L) or sodium acetate (0.92 g/L). The feed was kept at neutral pH (7.0). The SOB culture was developed using enrichments from SRB reactors (van Hille and Mooruth, 2014).

### 2.2 Reactor set-up and operation

**FIGURE 1:**
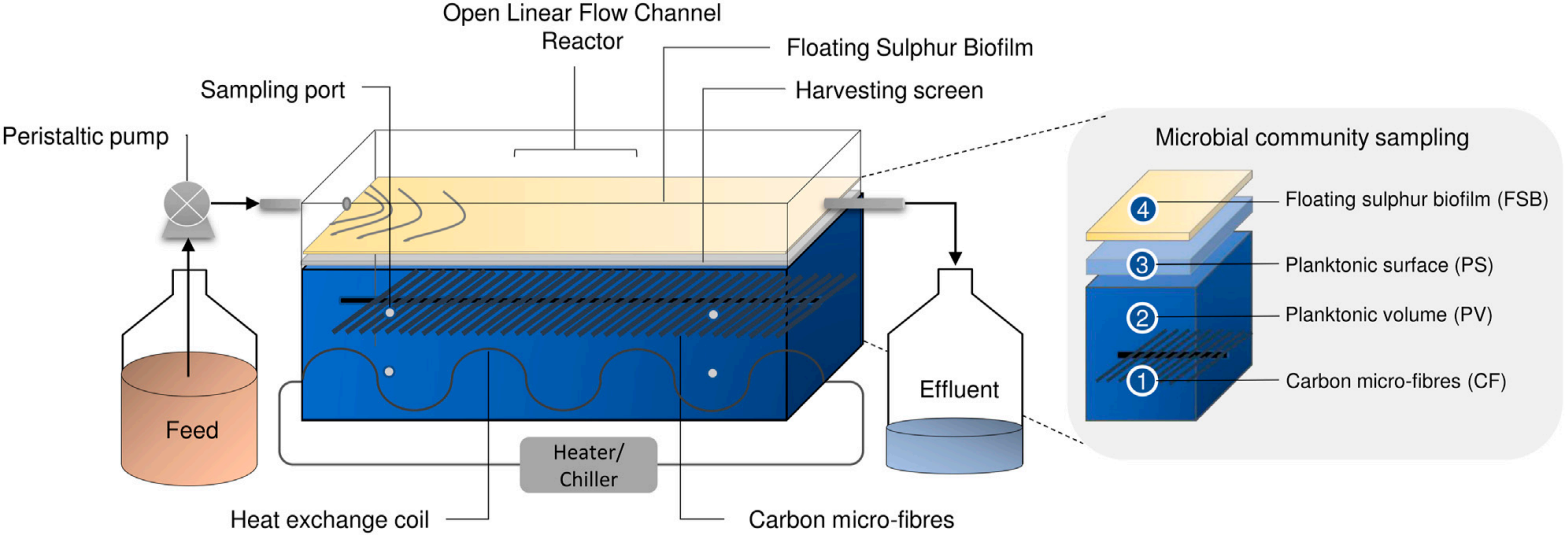
Schematic diagram showing the hybrid LFCR configuration and microbial ecology sampling of the CF, PV, PS, and FSB communities taken after operation at 30 and 10°C for 16S rRNA sequencing [adapted from Marais et al. (2020b)].

Laboratory-scale hybrid LFCRs, designed to enhance biomass retention and with a working volume of 2 and 8 L were previously described by Marais et al. (2020c). The 2 L reactors were constructed with internal dimensions of 250 mm (L) × 100 mm (w) × 150 mm (h), with an aspect ratio of (l/w) of 2.5. The 8 L reactor was designed with internal dimensions of 450 mm (L) × 200 mm (w) × 150 mm (h). The reactors included carbon microfibers as a microbial support matrix and a mesh screen, positioned just beneath the air-liquid interface, for biofilm recovery (Figure 1). They were fitted with a heat exchanger (4 mm ID) for temperature control. A peristatic pump continuously fed MPB medium into the reactors at a predetermined flow rate. Samples (2 ml) were taken daily from four sampling ports (Figure 1) positioned across the length and depth of the reactor. In addition, an effluent sample was collected at the exit port. The FSB was harvested intermittently by physical disruption of the biofilm and recovery of the accumulated settled biofilm by removing the screen.

### 2.3 Effect of temperature of fluid dynamics in the linear flow channel reactor

An understanding of the mixing profile within a reactor system is critical to ensure optimal functioning of the bioreactor and process performance. Inefficiencies in fluid mixing such as the development of dead zones or short-circuiting can be identified using tracer studies. A phenolphthalein dye tracer study, where the reactor was filled with a dilute sodium hydroxide solution and an acid solution was fed in, was used to evaluate the effect of temperature on the fluid dynamics within the LFCR. The acid neutralises the alkalinity, causing a colour change from pink to clear. The same procedure detailed by Marais et al. (2020a) was applied in the current study. The temperature of the bulk liquid in the reactor was regulated using a circulating water bath which pumped heated or cooled water through the internal heat exchanger (Figure 1). Temperature conditions assessed were 10, 15, 20, 25, and 30°C. The average bulk reactor volume temperature was checked and maintained using a digital thermometer. The hydrodynamic mixing profile has been shown to be conserved across both 2 and 8 L LFCR units across a range of HRTs (Marais et al., 2020a). Therefore, the study was only performed on the 8 L LFCR. The reactor was operated at a constant flow rate, equivalent to a 2 day HRT, and the tests were repeated three times. Photographic images were taken over the course of every experimental run and complete mixing time was recorded. Results were used to evaluate and describe the mixing dynamics occurring within the reactor Marais et al. (2020a).

### 2.4 Evaluating the effect of temperature on process performance

A 2 L lactate-fed reactor had been operated continuously for 1,137 days since the initial laboratory-scale demonstration (Marais et al., 2020a; Marais et al., 2020b). The 8 L lactate-fed and 2 L acetate-fed reactors were operated for approximately 488 and 524 days prior to the current investigation, respectively (Marais et al., 2020c). In this study, temperature was gradually decreased in increments of 5°C from 30 to 10°C. Initially, the reactors were operated at 30°C with a 2 day HRT (Marais et al., under review). After approximately 3 residence times (RTs) at a 2 day HRT (equivalent to 6 days of continuous operation), the sulphur-rich biofilm was physically disrupted, allowing the fragments to settle onto the harvest screen. The process was operated for an additional 3 RTs before the biofilm was disrupted again. The total biofilm accumulated over the combined 6 RTs (12 days) was recovered by removing the harvesting screen. Thereafter, the temperature was decreased to 25°C and the process was repeated. The total sulphate load and FSB harvesting regime was kept constant. This was to ensure consistency across the study, particularly for assessing the effect of temperature on sulphur recovery from the FSB and comparing reactor size and carbon source. The harvested sulphur biofilms were collected, dried at 60°C overnight, prior to elemental analysis. The selection of the 2 day HRT and biofilm disruption interval was informed by previous optimisation experiments (Marais et al., 2020b). These showed that, at 30°C, complete biofilm recovery occurred within 24 h of disruption and that if the disruption interval was extended beyond 3 HRTs the biofilm became so thick that oxygen mass transfer was impeded to the point where sulphide oxidation became limiting and a significant increase in aqueous sulphide concentration was detected in the effluent.

### 2.5 Analytical methods

The pH was measured using a Cyberscan 2500 micro pH meter and redox potential using a Metrohm pH lab 827 redox meter fitted with a Metrohm Redox platinum-ring electrode. Dissolved sulphide was quantified using the colorimetric N, N-dimethyl-p-phenylenediamine method (APHA, 2012). Residual sulphate was measured using the turbidimetric barium sulphate precipitation method (APHA, 2012). Volatile fatty acid (VFA) analysis was conducted using HPLC to quantify the feed and residual concentration of lactic, acetic, and propionic acids (Marais et al., 2020a). Elemental analysis of the FSB was determined using an Elementar Vario EL Cube Elemental Analyser.

### 2.6 Microbial community analysis

#### 2.6.1 Sampling and sequencing strategy

Samples for genomic DNA extraction were taken from discrete microenvironments that were identified to provide a complete coverage of the different microbial communities present within the hybrid LFCR (Figure 1) (Marais et al., under review). These included: 1) biomass attached to the carbon microfibers (CF), 2) planktonic cells from within the anoxic bulk volume (PV), 3) planktonic cells sampled just below the air-liquid interface (PS), and 4) the FSB.

#### 2.6.2 DNA extraction and Illumina® Miseq® 16S rRNA amplicon sequencing

Genomic DNA was extracted from a total of 24 selected samples using the NucleoSpin soil genomic DNA extraction kit (Machery-Nagel, Germany) as per manufacturers’ instructions. DNA purity and concentration were determined using a Nanodrop 2000 (Thermo Scientific). Samples were stored at −60°C and processed by Macrogen (South Korea) for library preparation, Illumina® MiSeq® sequencing, pre-processing, OTU clustering and taxonomic assignment. Briefly, dual-index barcoded V3-V4 region sequence libraries were generated by limited cycle polymerase chain reaction (PCR) to yield an approximately 460 bp amplicon, using the bacterial 16S rRNA gene oligonucleotide primers Bakt_341F and Bakt_805R (Mizrahi-Man et al., 2013). Amplification reactions were performed in a thermal cycler using an initial denaturation step at 95°C for 3 min followed by 25 cycles of denaturation at 95°C for 30 s, annealing at 55°C for 30 s, elongation at 72°C for 30 s, and a final extension step at 72°C for 4 min. Amplicon libraries were sequenced on an Illumina® MiSeq® sequencer to yield 300 bp paired-end reads. Raw sequencing reads have been deposited to the NCBI’s Sequence Read Archive (SRA) and is accessible through the BioProject accession number PRJNA818253.

#### 2.6.3 Metagenomic ASV assignment and taxonomy alignment

The 16S rRNA amplicon gene sequences were analysed using the Divisive Amplicon Denoising Algorithm 2 (DADA2) pipeline as described by Callahan et al. (2016). Briefly, the raw sequencing data were filtered; the adapter, barcode and primer sequences were removed; and the ends of amplicon sequences with high numbers of errors were trimmed. The amplicons were denoised based on a model of the sequencing errors and paired-ends were merged. Chimaeras were removed and sequences clustered into Amplicon Sequence Variants (ASVs). Taxonomy assignment of resulting ASVs were performed using the SILVA reference database (v132) (Quast et al., 2013).

#### 2.6.4 Metagenomic statistical analysis

Statistical and visual analysis was performed using MicrobiomeAnalyst (Dhariwal et al., 2017). Alpha diversity measurements were determined using the Shannon and Simpson diversity metrics. Beta diversity was evaluated through the Bray-Curtis dissimilatory metric and non-metric multidimensional scaling (NMDS) ordination method. Hierarchical clustering was performed using Clustvis (Metsalu and Vilo, 2015) to evaluate the distribution of ASVs across reactor samples based on Eluclicean distance and Ward linkage. ASVs were clustered using correlation and average. Row centring was applied in combination with data transformation and unit variance scaling.

## 3 Results and discussion

### 3.1 Effect of temperature on fluid mixing dynamics in the hybrid linear flow channel reactor

The hydrodynamic mixing profile in the LFCR was consistent at temperatures between 30 and 15°C, with the initial acid front sinking to the bottom of the reactor before moving in a horizontal direction toward the effluent port (Supplementary Figure S1). The mixing regime was governed primarily by advective and diffusive transport. The relatively slow linear velocity and absence of turbulent mixing ensures minimal disturbance at the surface of the reactor. Mooruth, (2013) calculated Reynolds numbers of less than 30 for similar reactors, indicating very low levels of turbulence. This is critical to ensure suitable conditions to support the development of a floating sulphur biofilm at the air-liquid interface. The hydrodynamic profile between 15 and 30°C was consistent with previous studies conducted at 28°C (Marais et al., 2020a).

**FIGURE 2:**
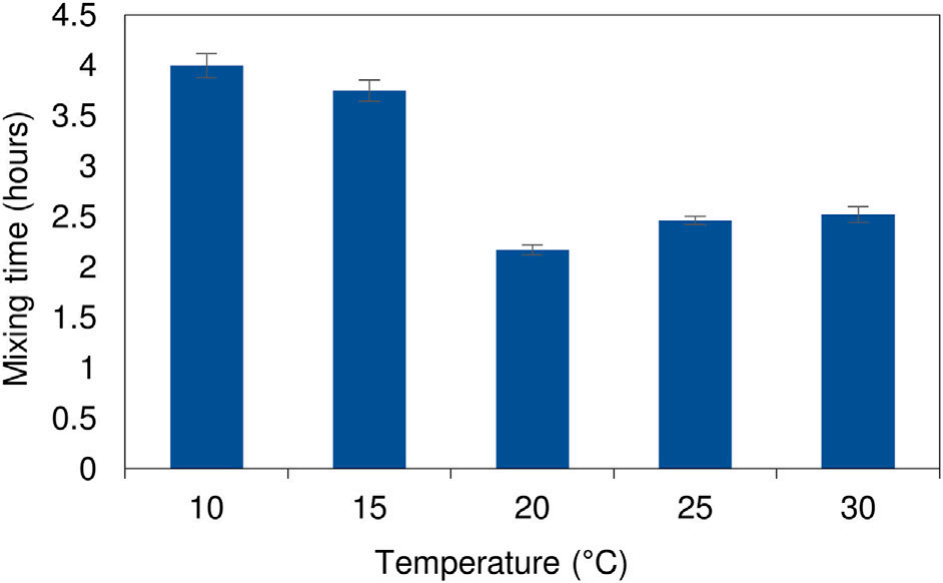
Complete mixing times of the dye tracer study as a function of temperature within the 8 L reactor operated at a 2 day HRT. The experimental runs were performed in triplicate with the standard deviation shown by the error bars.

At low temperatures (10 and 15°C) the mixing pattern varied with a zone of clearing initially forming near the surface of the bulk reactor volume (Supplementary Figure S2). This was attributed to the temperature differential affecting the relative density between the inlet feed and the bulk liquid. After 145 min of continuous operation the initial zone of clearing remained confined as a narrow zone across the air-liquid interface. By contrast, at ambient temperature (25°C), complete mixing had occurred by this stage. The lower rate of diffusive mixing led to an increase in complete mixing times over the colder temperature range (10 and 15°C). The experiments conducted at elevated temperatures showed increased diffusive mixing during the initial stages (Supplementary Figure S3). While most of the reactor volume was well mixed within a relatively short period of time, indicated by disappearance of the pink colour, several dead zones were observed at the base of the reactor. These took longer to reach equilibrium conditions. At lower temperatures (10 and 15°C), there was a temperature differential between the feed (at ambient temperature) and the cooled bulk reactor volume. In all cases, attempts were made to minimise the temperature differential by adjusting the feed reservoir temperature to that of the reactor. The alterations had minimal impact on the outcome of the results. The slow flow rate resulted in the feed gaining or losing heat on pumping from the reservoir to the reactor. Although the increase in complete mixing times were longer at the lower temperature range (<20°C), they were still considerably shorter (<4 h) (Figure 2) than the operating HRT of 2 days. Further evaluation of the process performance confirmed that there was little deviation in measured concentration across sampling ports along the length and depth of the reactor, indicating a relatively well mixed system with no signs of short-circuiting. The differences between the high and low temperature range did not have an impact on the overall functioning of the hybrid LFCR.

### 3.2 Effect of temperature on biological sulphate reduction performance

Optimal pH and redox conditions for sulphate reduction were maintained over the course of the study. The redox potential was relatively stable in all three reactors with an average of −363 ± 13, −365 ± 11, and −362 ± 29 mV for the 2 L lactate-fed, 8 L lactate-fed and 2 L acetate-fed reactors, respectively. The pH remained relatively stable within all three reactors over the duration of the study. However, there were distinct differences observed across reactor systems. In the 2 L lactate-fed and 8 L lactate-fed reactors, the average pH of the reactor samples ranged between 7.0 and 7.5, while the pH of the effluent samples was higher. For most of the study, the 8 L lactate-fed reactor maintained a pH similar to the feed (pH 7.0) when operated between 20 and 10°C, with little fluctuation even after biofilm disruption and harvesting. This was different to previous experiments, where the reactor pH increased noticeably after biofilm disruption, coinciding with a rapid decrease in sulphide concentration.

The reactor and effluent pH profiles measured in the 2 L acetate-fed reactor (Supplementary Figure S4) were higher than both lactate-fed reactors and ranged between 7.2 and 8.0. This can be attributed to the oxidation of acetate as a carbon source generating more alkalinity than the partial oxidation of lactate. In addition, the partial oxidation of lactate generates a mole of acetate for every mole of lactate consumed with the residual acetate concentration typically above 10 mmol/L, thus lowering the pH within the lactate-fed reactors (Eqs 2, 3; Oyekola et al., 2009). The effluent pH was consistently higher than the pH of the reactor samples for all three reactor systems. This was attributed to partial sulphide oxidation at the surface, whereby hydroxyl ions are released during the formation of elemental sulphur. These findings were consistent with previous investigations (Marais et al., 2020a; Marais et al., 2020b; Marais et al., 2020c).

$$3\;\mathrm{Lactate}\;\to \mathrm{acetate}+2\;\mathrm{propionate}+\mathrm{HC}O_{3}^{-}+\;H^{+}$$ (1)

$$2\;\mathrm{Lactat}e^{-}+SO_{4}^{2+}\;\to HS^{-}+2\;\mathrm{Acetat}e^{-}+2\;\mathrm{HC}O_{3}^{-}+H^{+}$$ (2)

$$\mathrm{Acetat}e^{-}+SO_{4}^{2-}\;\to HS^{-}+2\;\mathrm{HC}O_{3}^{-}$$ (3)

There was a shift in the VFA concentration profile over time in response to decreasing temperature (Supplementary Figure S4). At 30°C, complete lactate utilisation occurred in both 2 and 8 L lactate-fed reactors. The increase in residual acetate, concomitant with the conversion of sulphate, indicated that incomplete lactate oxidation was the dominant metabolic pathway (Eq. 2). A consistent generation of propionate indicated that a fraction of the lactate was fermented, rather than oxidised, in both lactate-fed reactors (Supplementary Figure S4; Eq. 1). A decreasing trend in both volumetric lactate utilisation rate and production rate of acetate and propionate were observed as temperature decreased from 30 to 10°C in both the 2 and 8 L reactors. At 10°C, lactate utilisation decreased to 73 ± 14 and 71 ± 16% in the 2 and 8 L lactate-fed reactors, respectively. This corresponded to a decrease in volumetric lactate utilization rate from 0.228 to 0.141 mmol/L.h and 0.228 to 0.162 mmol/L.h within the 2 and 8 L lactate-fed reactors, respectively.

The effect of temperature on acetate utilisation could not be accurately determined, due to the production of acetate by microbial conversion of the fermentable fraction of the yeast extract. In the acetate-fed reactor, this led to the measured residual acetate concentrations being higher than those in the inlet feed, particularly during operation at 30°C (Supplementary Figure S4E). The discrepancy in acetate utilisation and production rates was observed in all three reactors with excess acetate generation also occurring within the lactate-fed reactors. Further, stoichiometric analysis based on feed lactate concentration revealed that more acetate was generated than theoretically possible *via* lactate fermentation and/or incomplete sulphate reduction (Eqs 1, 2), owing to its production from yeast extract (Marais, et al., 2020b).

**FIGURE 3:**
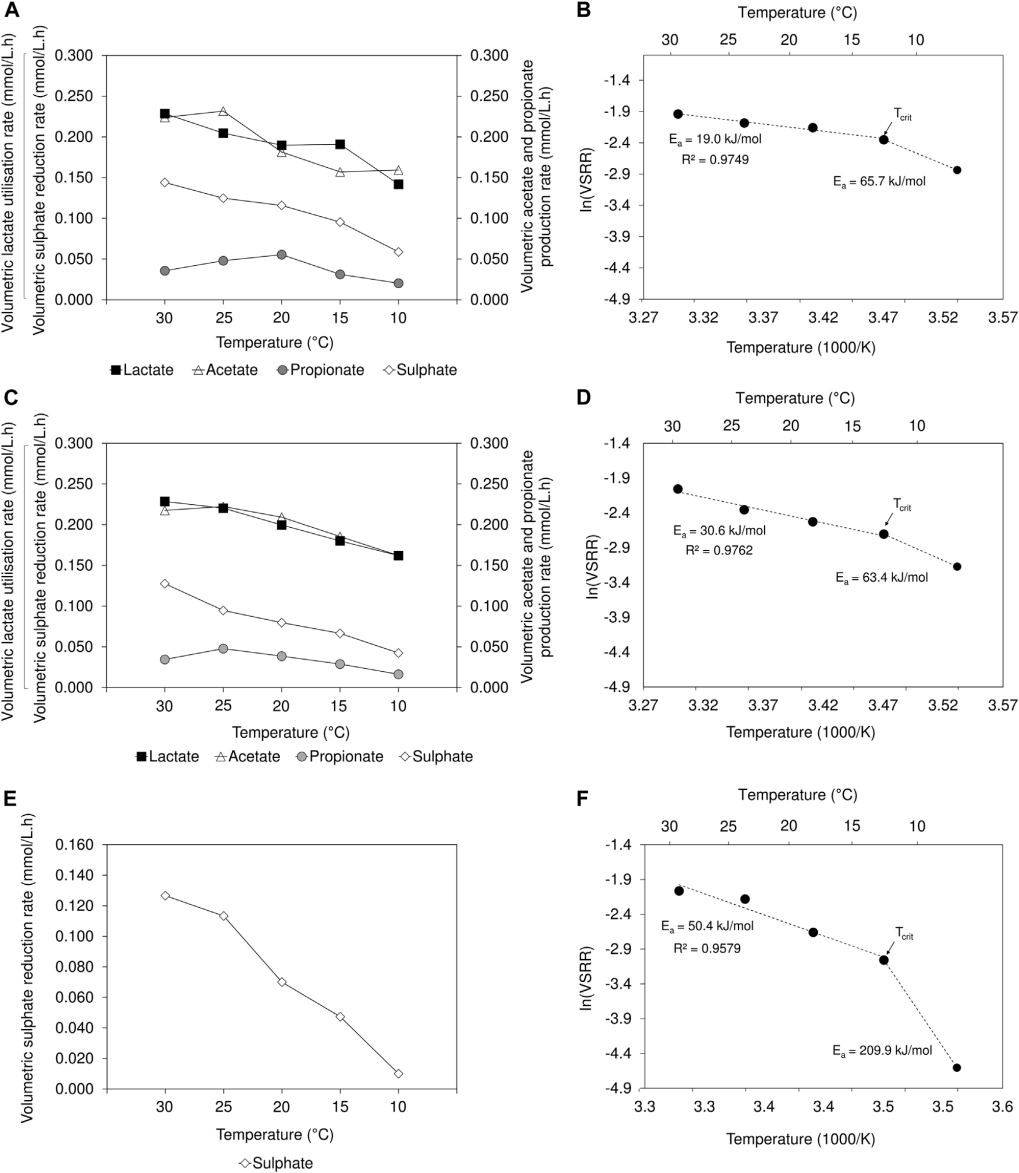
Temperature dependency on VSRR, VFA utilisation/production rates in the **(A)** 2 L lactate-fed, **(C)** 8 L lactate-fed and **(E)** 2 L acetate-fed reactors. Arrhenius plots showing temperature dependency of VSRR, showing the E_a_ values for the linear range between 30 and 15°C as well as the deviation at 10°C in the **(B)** 2 L lactate-fed **(D)** 8 L lactate-fed and **(F)** 2 L acetate-fed reactor. The indicated T_crit_ represents the critical temperature, below which microbial activity is significantly reduced.

A decrease in volumetric sulphate reduction rates (2 L lactate-fed: 0.144 to 0.059 mmol/L.h; 8 L lactate-fed: 0.128 to 0.042 mmol/L.h; 2 L acetate-fed: 0.127 to 0.010 mmol/L.h) and sulphate conversion efficiency (2 L lactate-fed: 66% to 27%; 8 L lactate-fed: 61% to 20%; 2 L acetate-fed: 61% to 5%) was observed over the temperature range between 30 and 10°C (Figure 3; Supplementary Table S1). The results were supported by previous findings where an increase in VSRR and sulphate conversion efficiency were associated with increased temperatures (Moosa et al., 2005; Al-zuhair et al., 2008). The decrease in lactate utilisation and production of acetate and propionate, concomitant with the decline in VSRR, indicated that the activity of both SRB and fermentative microbial populations was adversely affected in the lactate-fed reactors. In the 2 L acetate-fed reactor, a sulphate conversion of 61% and 54% was achieved at 30 and 25°C, respectively. The performance was comparable to that achieved in the lactate-fed reactors. At 20°C, the 2 L lactate-fed reactor performed best and maintained a sulphate conversion >50%. In the 2 L acetate-fed and 8 L lactate-fed reactors, sulphate conversion decreased to 34% and 38%, respectively. In the acetate-fed reactor, operation at 10°C led to a considerable decrease in sulphate reduction activity, only achieving a conversion of 5%. This was four to five times lower than the 27% and 20% sulphate conversion achieved in the 2 and 8 L lactate-fed reactors at 10°C. Despite the decline in process performance within all three reactors, BSR activity was not completely suppressed at 10°C.

#### 3.2.1 Temperature dependence of sulphate reduction rates

To better understand the effects of temperature on sulphate reduction, the Arrhenius Eq. 4 was applied to model the temperature dependence of VSRR in the hybrid LFCR as follows (Sawicka et al., 2012; Robador et al., 2016):

$$\ln\left(k\right)=\ln\left(A\right)+\left(\frac{-E_{a}}{R}\times \frac{1}{T}\right)$$ (4)

where *E*_*a*_ is the activation energy (J/mol), *k* is the rate of sulphate reduction (mmol/L.h), *A* is the Arrhenius constant, *R* is the gas constant (8.314 J/K.mol) and *T* is the absolute temperature (K). The activation energies were calculated from the linear range of the plot of experimental VSSR data as a function of the inverse temperature (1,000/T) (Figure 3).

In microbial systems, E_a_ estimates are commonly interpreted to reflect the temperature response of the rate-limiting step in a physiological process such as enzymatic catalytic conversions. The activity of an efficient enzyme with low temperature dependence will yield a low E_a_ (Robador et al., 2016). For a mixed microbial community, E_a_ values are not representative of a single sulphate reducing population but reflect the response of the community. Studies have shown that E_a_ values calculated from pure SRB cultures were similar to those of a mixed SRB community (Robador et al., 2016). This suggests that many SRB have similar responses to changes in temperature. Therefore, the E_a_ value is a suitable estimate that can be applied to describe the temperature sensitivity of the SRB community within the hybrid LFCR process.

In all three reactors, the VSRR-temperature relationship (Figure 3) remained linear (2 L lactate-fed: *R* ^2^ = 0.98; 8 L lactate-fed: *R* ^2^ = 0.98; and 2 L acetate-fed: *R* ^2^ = 0.96) between 30 and 15°C. As temperature decreased, there was an exponential reduction in the reaction rates; the magnitude of which depended on the activation energy. In the current study, the calculated E_a_ values over the temperature interval 30 to 15°C were 19.0, 30.6, and 50.4 kJ/mol in the 2 L lactate-fed, 8 L lactate-fed and 2 L acetate-fed reactors, respectively. The difference in E_a_ value between the 2 and 8 L lactate-fed reactors is more likely due to the fact that the 2 L reactor had been operated for significantly longer prior to the start of this experiment, facilitating greater biomass accumulation and the associated reduced contribution of the planktonic community, rather than the difference in reactor geometry. The highest E_a_ was determined in the acetate-fed reactor (1.6 to 2.6 fold that of the lactate fed reactor), showing a greater sensitivity of acetate-utilising microorganisms to low temperature in the range 30 to 15°C.

Temperatures that result in deviation from the linear range of the Arrhenius plot (Figure 3) are known as stress inducing temperatures. This deviation was observed between 15 and 10°C for all three reactors. As a result, the VSRR decreased rapidly, exhibiting a stronger temperature dependency with higher E_a_ values (2 L lactate-fed: 65.7 kJ/mol; 8 L lactate-fed: 63.4 kJ/mol; 2 L acetate-fed: 209.9 kJ/mol) determined between 15 and 10°C. Again, these findings demonstrate the greater sensitivity of the acetate culture to low temperatures. The temperature at which the two lines (linear range and deviation) intersect is defined as the critical temperature (T_crit_) (Figure 3). The T_crit_ for bacterial growth at low temperature is used to identify the transition between optimal and sub-optimal thermal activity ranges (Robador et al., 2016). Operation below T_crit_ results in significant decrease in microbial activity, attributed to growth inhibition within a community. Below the optimum growth temperature, the affinity to sequester substrates decreases (Nedwell, 1999). This is consistent with the increase in residual lactate observed upon decreasing from 15 to 10°C, indicating reduced lactate utilisation within both lactate-fed reactors (Supplementary Figure S4).

The linear Arrhenius range defines the temperature to which a community or microorganism is optimally adapted. In this study, temperatures exceeding 30°C were not evaluated, thus the T_max_ was not determined. However, most mesophilic SRB grow optimally at temperatures between 25 and 40°C (Moosa et al., 2005; Sheoran et al., 2010; Hao et al., 2014). The observed trend in relationship between microbial activity and temperature results from direct or indirect effects. At low temperatures, direct effects include reduced growth rate and enzyme activities as well as the alteration of cell composition, while indirect effects are associated with the solubility of solute molecules, diffusion of nutrients and osmotic effects on membranes and cell density (Bisht, 2010).

### 3.3 Effect of temperature of sulphide oxidation performance

Volumetric sulphide oxidation rates (VSOR) were determined using the theoretical sulphide (expected) generated and the average sulphide measured within the reactor samples and final effluent. Mooruth (2013) operated the LFCR as a sealed unit, with a lid and silicon gasket. The headspace was continuously flushed with air that was then passed through a caustic sulphide scrubber. The results showed that loss of gaseous hydrogen sulphide across the air liquid interface was negligible (<0.5%), even when the biofilm was incomplete, most likely due to the lack of turbulence in the bulk liquid. Subsequent experiments were performed using uncovered reactors. There was no detectable sulphide odour and the absence of detectable concentrations of hydrogen sulphide above the reactor surface was confirmed using a portable hydrogen sulphide detector (Dräeger).

**FIGURE 4:**
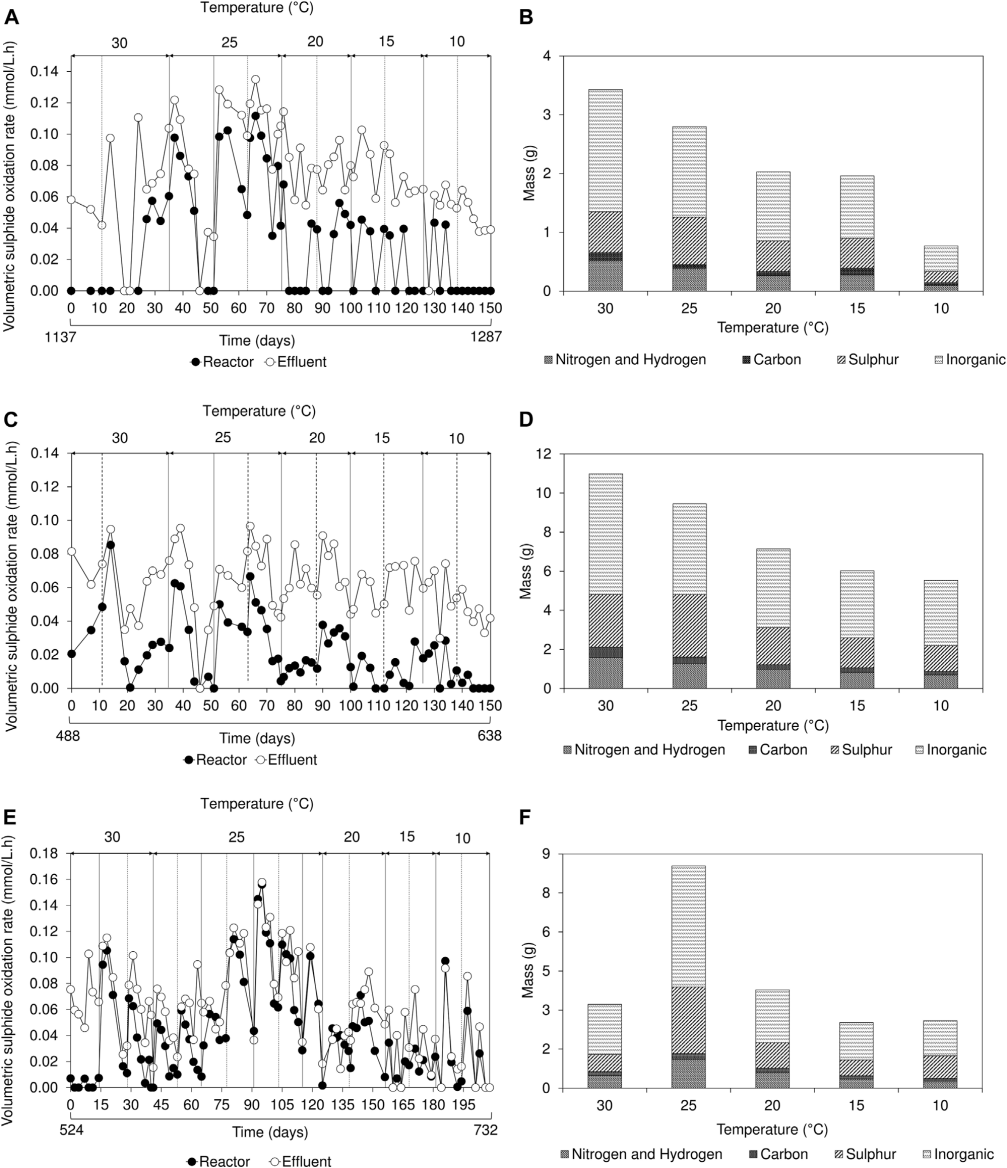
Effect of temperature on **(A,C,E)** volumetric sulphide oxidation rates over time and **(B,D,F)** floating sulphur biofilm recovery showing the elemental composition of inorganics, sulphur, carbon, nitrogen, and hydrogen based on the amount (grams) of biofilm harvested, in the **(A,B)** 2 L lactate-fed, **(C,D)** 8 L lactate-fed and **(E,F)** 2 L acetate-fed reactors. The inorganic refers to the fraction that was not determined by CHNS analysis.

During initial operation, once a biofilm disruption occurred, aqueous sulphide concentration rapidly decreased. After 24 h, sulphide concentration gradually increased over time as the FSB regenerated at the air-liquid interface (Supplementary Figure S5). This distinctive trend in sulphide concentration, a well-defined characteristic of the hybrid LFCR process (Marais et al., 2020b), was observed in all three reactors during operation at 30 and 25°C. The decrease in sulphide concentration following biofilm disruption, corresponded to an increase in VSOR; this reached a maximum, then decreased with extended time. As the FSB develops at the air-liquid interface, it impedes oxygen mass transfer into the bulk volume of the reactor and inhibits sulphide oxidation, thus necessitating the periodic disruption of the biofilm. The removal of the biofilm at the surface results in an increase in oxygen mass transfer into the bulk volume, allowing sulphide oxidation to proceed. In this study, during operation between 25 and 10°C, the trend in decreasing sulphide concentration after biofilm disruption was not observed (Supplementary Figure S5). Instead, the sulphide concentration remained relatively stable, gradually decreasing as temperature was reduced. Reduced sulphide oxidation kinetics was particularly evident within the bulk volume of the lactate-fed reactors (Figures 4A,C). The low VSOR recorded within the reactor samples, even in the presence of sufficient sulphide, suggests that SOB activity was adversely affected at low temperatures.

The estimated VSOR, based on the expected sulphide concentration, revealed that sulphide oxidation in the effluent samples was consistently higher than compared to the reactor bulk volume samples (Figure 4). This suggested that sulphide oxidation was confined predominantly to the surface of the reactor at the low temperatures and can be attributed to a combination of oxygen availability as well as the variability in temperature experienced at the surface. In the hybrid LFCR, the air-liquid interface was exposed to both the regulated temperature in the bulk volume and ambient temperature from the surrounding environment.

#### 3.3.1 Effect of temperature on sulphur recovery from biofilm

**FIGURE 5:**
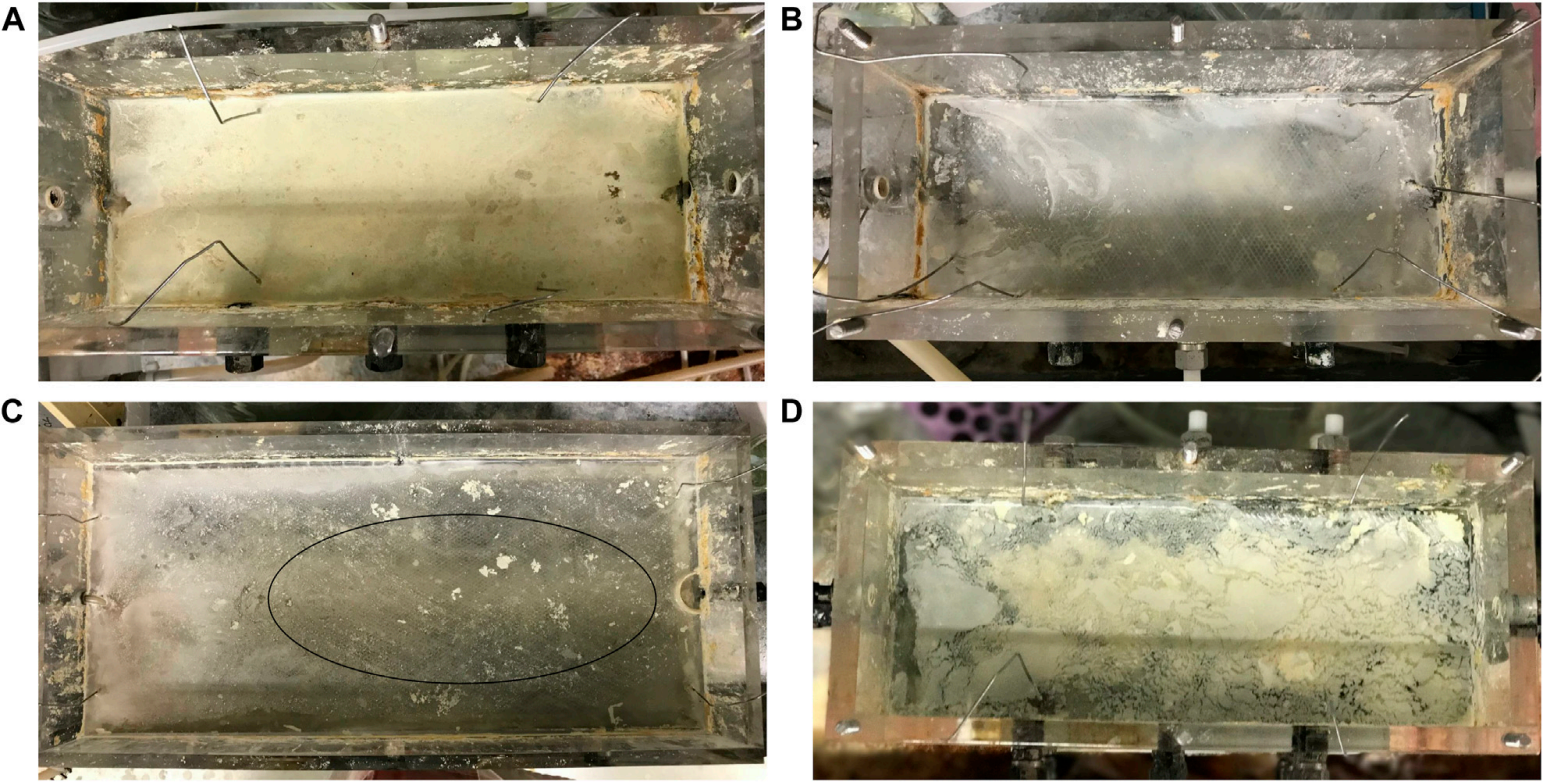
Photographs of the floating sulphur biofilm formation after disruption while operated at 2 day HRT showing the **(A)** 2 L lactate-fed reactor at 30°C after 2 days (well-developed biofilm), **(B)** 2 L lactate-fed reactor at 10°C after 2 days (poor formation), **(C)** 8 L lactate-fed reactor at 10°C after 2 days (circle indicates clear zone), and **(D)** 2 L acetate-fed reactor at 25°C after 5 days (premature disruption).

FSB recovery decreased in the 2 and 8 L lactate-fed reactors, from 4.9 to 1.1 g and 15.7 to 7.9 g respectively, after decreasing temperature from 30 to 10°C (Figure 4). This led to a reduction in overall biosulphur recovery (Supplementary Table S1). While this suggests that partial sulphide oxidation and sulphur recovery *via* the FSB were adversely affected by the decrease in temperature, the decline in BSR was also an important factor. In the hybrid LFCR, BSR activity dictates the amount of sulphide available for partial sulphide oxidation. The extent of biofilm formation under different operating conditions is shown in Figure 5. The rate of FSB development was slower at reduced temperature. In addition, the structural integrity of the FSB was compromised, often disintegrating into smaller fragments that washed out in the effluent stream. When operated at 30°C (Figure 5A), a well-established biofilm covered the entire surface of the 2 L lactate-fed reactor within 48 h. In contrast, at 10°C (Figures 5B,C) in both the 2 and 8 L lactate-fed reactors, the biofilm was less developed and had a consistency that resembled the initial sticky/thin stage of development at 2 days. In the 2 L acetate-fed reactor at 25°C, the biofilm collapsed without intentional disruption, ahead of schedule (Figure 5D). This resulted in the formation of a new floating sulphur biofilm outside of the studies’ structured interval period and led to a larger amount of biofilm to be recovered at 25°C (Figure 4).

Analysis of the FSB revealed a shift in elemental composition in response to temperature change (Figure 4). Decreasing temperature from 30 to 25°C resulted in a 10% increase in sulphur composition across all three reactors (2 L lactate-fed: 20%–29%; 8 L lactate-fed: 24%–34%; 2 L acetate-fed: 21%–30%). A further decline from 25 to 10°C, decreased the elemental sulphur content by 4% and 8% in the 2 and 8 L lactate-fed reactors, respectively. A major component of the FSB was determined to be an inorganic crystalline mineral precipitate that accounted for 56 ± 4, 57 ± 2, and 55 ± 3% of the FSB in the 2 L lactate-fed, 8 L lactate-fed, and 2 L acetate-fed reactors, respectively. The crystals embedded within the sulphur biofilm were later identified through SEM-EDS as struvite. Struvite is comprised of equimolar concentrations of magnesium (Mg), ammonium (NH_4_), and phosphorus (P). The modified Postgate B medium (MPB) used in this study has been used as a nutrient rich medium for the cultivation of SRB and to evaluate biological sulphate reduction in many published studies. The majority of the sulphate is added as magnesium sulphate, rather than sodium sulphate, to reduce the potential inhibition by monovalent cations. In actual mine-impacted water most of the sulphate is derived from pyrite oxidation, so the magnesium concentration would be substantially lower. The MPB also contains relatively high concentrations of ammonium and phosphate that would promote the formation of struvite, by evaporative crystallisation at the air-liquid interface and potentially biomineralisation. The biomineralisation of phosphate minerals have been demonstrated to be a by-product of metabolism of specific microorganisms. A study by Soares et al. (2014) reported a selection of bacteria capable of biologically mediating the formation of struvite (biostruvite) when incubated in sterile sludge dewatering liquors.

Mean elemental sulphur recovery *via* the FSB, determined based on FSB-S (grams of sulphur recovered from the biofilm) and the total amount of converted sulphide-S (grams of sulphur), was 30 ± 4.6 and 33 ± 6.0% (mean ± SD) in the 2 and 8 L lactate-fed reactors, respectively, over the range of temperatures evaluated. In the 2 L acetate-fed reactor, sulphur recovery at 30 and 25°C were 29% and 65%, respectively. The high sulphur recovery at 25°C was consistent with the higher biomass harvested due to premature disruption and additional FSB regeneration. These results highlight the importance of biofilm management and frequency of disrupting the biofilm to achieve maximum sulphur recovery *via* the FSB.

In the 2 L acetate-fed reactor, sulphur recovery at 15 and 10°C could not be determined. This was due to a combination of minimal sulphate conversion (5%), relatively higher biomass recovery than expected and discrepancies observed between the expected and measured aqueous sulphide concentrations. Low sulphide production and poor FSB formation in the acetate-fed reactor led to uncontrolled oxygen mass transfer at the air-liquid interface, facilitating complete oxidation of elemental sulphur to sulphate. In sulphide oxidation processes the molar ratio of sulphide to oxygen is a critical operating parameter to ensure elemental sulphur production. Since the supply of oxygen into the hybrid LFCR is not regulated, the floating biofilm plays a pivotal role in reducing oxygen mass transfer. Therefore, it is critical that there is active BSR and sufficient aqueous sulphide in the bulk liquid when the biofilm is disrupted to ensure that any oxygen dissolving into the bulk volume while the biofilm regenerates is consumed. Failure to ensure this will lead to an increase in redox potential, inhibition of the SRB and reactor failure (Mooruth, 2013).

The fraction of converted sulphide (gap-S) that could not be accounted for by recovery through the FSB was approximately 70% in the lactate-fed reactors (Supplementary Table S1). There was little increase in residual sulphate measured between the reactor samples and final effluent (Supplementary Figure S5), so complete oxidation to sulphate was negligible, and insignificant loss of sulphide as gaseous hydrogen sulphide. Therefore, the gap-S fraction was predominantly made up of colloidal sulphur particles suspended in solution and fragments of biofilm that were released into the effluent overflow. This was confirmed by the significant build-up of sulphur deposit within the effluent pipe. In addition, due to the hydrophilic nature of biological produced sulphur, the physical disruption of the FSB between harvesting resulted in the dispersion of some sulphur into the liquid phase. These findings were consistent with similar investigations performed on the hybrid LFCR (Marais et al., 2020a; Marais et al., 2020b). The elemental sulphur can be recovered downstream through sedimentation or centrifugation which are commonly employed in commercial sulphide oxidation processes (Syed et al., 2006; Cai et al., 2017).

### 3.4 Effect of temperature on microbial community dynamics

#### 3.4.1 Microbial community diversity

High-throughput 16S rRNA amplicon sequencing of 24 samples collected from four discrete microenvironments within the three reactors during operation at 30 and 10°C was successfully performed. The number of ASVs detected in each sample ranged between 60 and 171 and the average read count per sample was approximately 35,184. The bacterial richness and diversity estimates (Shannon and Simpson) were calculated for each sample (Supplementary Table S2). Higher ASV counts were observed at 10°C than at 30°C across reactor samples (CF, PS, PV, and FSB). Furthermore, Shannon indices were on average higher at 10°C, indicating a higher microbial diversity while the lower values observed at 30°C may suggest that the microbial communities were more defined. This is likely a consequence of extended operation as a continuous flow through system, with relatively stable operating conditions, resulting in the selection of a stable microbial community dominated by a small number of prominent taxa that are more tolerant of high sulphide concentrations. Changes in environmental conditions can lead to ecological shift resulting in a diverse mix of taxa where other species that were below the threshold for analysis can become more dominant.

**FIGURE 6:**
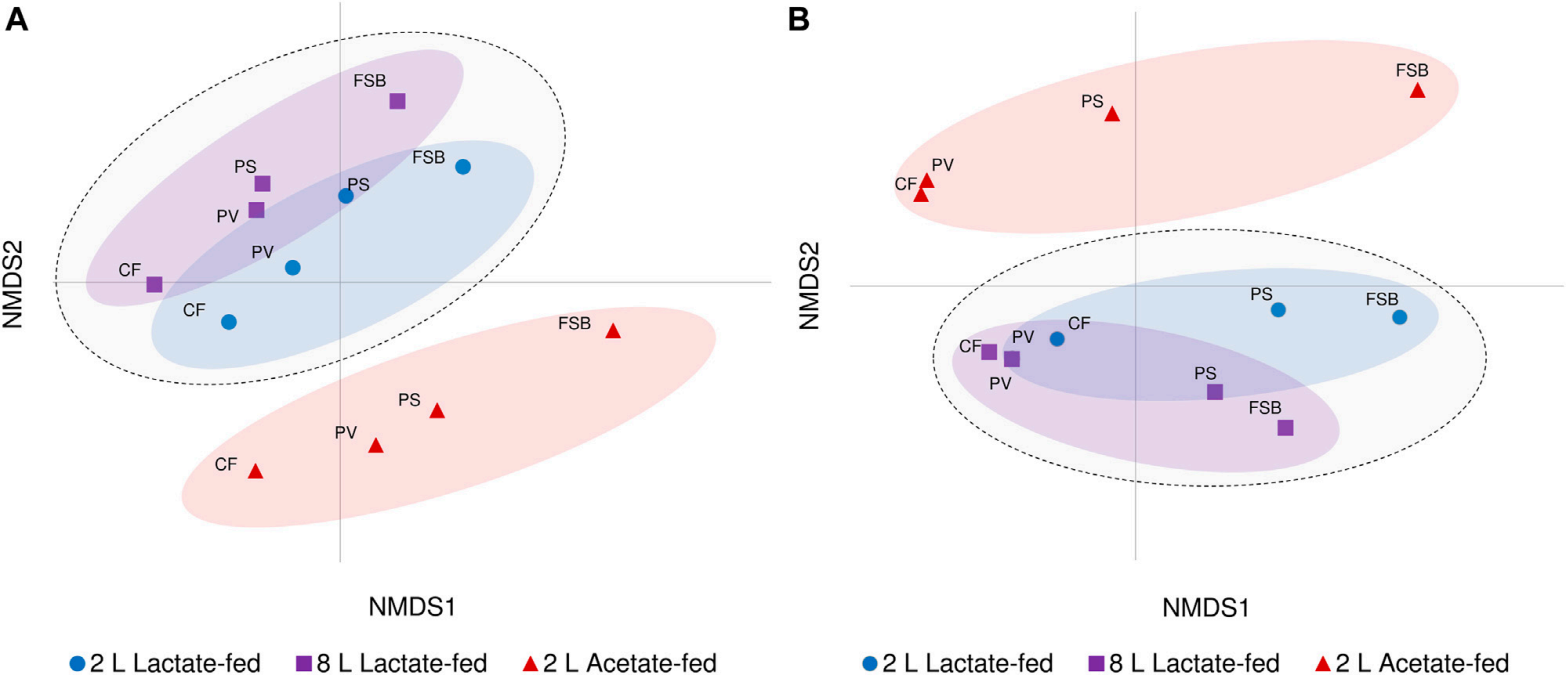
Non-metric multidimensional scaling (NMDS) ordination plot visualisation of beta diversity showing differences in microbial communities (CF, PV, PS, and FSB) in the 2 L lactate-fed, 8 L lactate-fed, and 2 L acetate-fed reactors at **(A)** 30°C and **(B)** 10°C.

Beta diversity analysis through NMDS ordination revealed the relationship and variability between microbial communities based on reactor geometry, carbon substrate and temperature (Figure 6). Samples derived from both lactate-fed reactors clustered together and were distinctly separate from samples associated with the 2 L acetate-fed reactor. Although a similar ordination of clusters based on the different reactors were preserved, divergence between discrete microbial communities at different temperature conditions was observed. The CF and PV communities clustered more closely together at 10°C. The PS communities which represented the transition phase between the anoxic bulk volume and FSB, were closely linked to the PV communities at 30°C. After a shift in community composition at 10°C, the PS communities were separate from the anoxic bulk volume communities (CF and PV) and more closely positioned with the FSB communities (Figure 6). The clear distinction between lactate- and acetate-fed reactors demonstrates the importance of the choice of carbon substrate as a key environmental parameter in shaping microbial community composition. Furthermore, the niche chemical microenvironments (anoxic and oxic zones) and microbial habitat (sessile and planktonic) within the hybrid LFCR facilitated the development of distinctly different microbial communities.

#### 3.4.2 Microbial community compositional shift at phylum level

**FIGURE 7:**
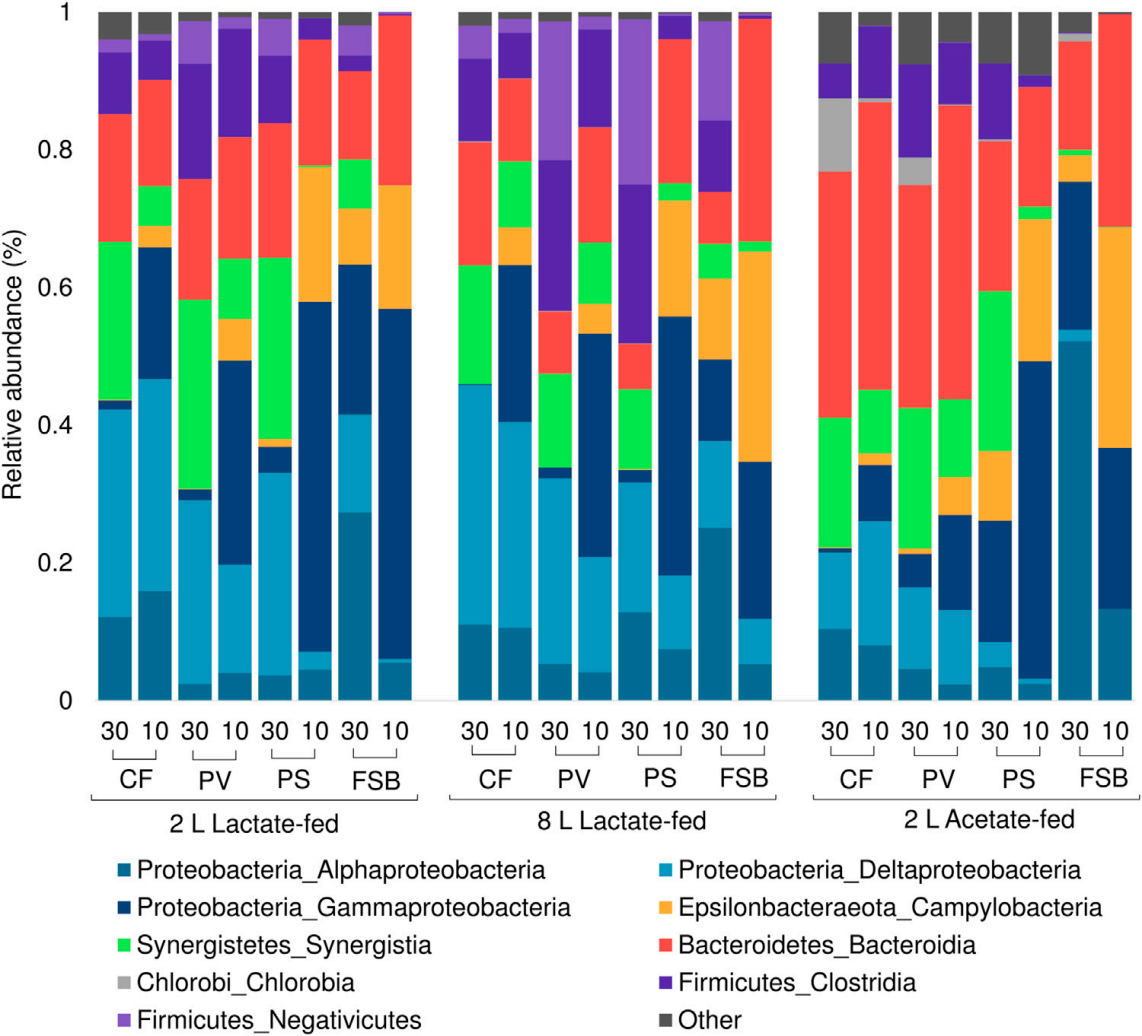
Taxonomic classification at phylum and class level showing the compositional shift in relative abundance of dominant affiliated sequences in the CF, PV, PS, and FSB microbial communities as a function of decreasing temperature from 30 to 10°C in the 2 L lactate-fed, 8 L lactate-fed, and 2 L acetate-fed reactors.

The microbial communities derived from discrete microenvironments within the anoxic bulk reactor volume and FSB comprised of five major phyla including Proteobacteria, Bacteroidetes, Firmicutes, Synergistetes, and Chlorobia (Figure 7). Chlorobia was only detected at high abundance within the 2 L acetate-fed reactor. A shift in microbial community composition in response to temperature change by cooling from 30 to 10°C led to an increase in the relative abundance of Gammaproteobacteria and Epsilonbactereaota with a concomitant decrease in Alphaproteobacteria, Synergistetes, Firmicutes, and Deltaproteobacteria across all three reactor systems. Deltaproteobacteria, which represents the largest phylogenetic group of known SRB, decreased in relative abundance, particularly within the planktonic communities (PS and PV) and FSB, suggesting increased washout owing to reduced growth rates. Interestingly, Deltaproteobacteria in the CF communities increased in relative abundance within the 2 L lactate- and acetate-fed reactors with a slight decrease observed in the 8 L lactate-fed reactor.

Both lactate-fed reactors shared a similar community structure and shift in response to temperature change. In the 2 L acetate-fed reactor the bulk volume communities (CF, PV), were different to the lactate-fed reactors with a lower prevalence of Deltaproteobacteria and higher abundance of the phyla Bacteroidetes. At a phylum level the FSB and PS community structures were relatively consistent across all three reactor systems. This was consistent with a previous investigation that evaluated the microbial community dynamics within the reactors as a function of HRT (Marais et al., TBA). The different source of carbon substrate did not influence the microbial composition of the FSB communities between the reactors. This suggests that the heterotrophs in the FSB can grow on acetate alone, consistent with previous observations by Mooruth (2013) in a two stage reactor system.

The shift in microbial community composition observed in the PS samples can be attributed to the structural integrity of the FSB. During operation at 30°C, a well-established FSB (Figure 5A) created a barrier to oxygen mass transfer across the air-liquid interface. This led to the conditions within the PS zone just below the FSB to be more representative of the anoxic bulk reactor volume. In contrast, during operation at 10°C the poor development and fragmentation of the FSB (Figures 5B,C) is likely to have led to unimpeded oxygen ingress across the air-liquid interface. The change in local physicochemical conditions at the PS zone became less favourable for the growth of obligate anaerobic phyla such as Deltaproteobacteria, Synergistetes, and Firmicutes, all of which exhibited a drastic decrease in relative abundance within the PS and FSB communities. In addition, the poorly formed biofilm often fragmented into the PS zone and eventually settled onto the harvesting screen. The continued dispersion into the lower regions of the reactor increased the relative abundance of microorganisms that were found to predominate at the oxic zone. This is likely to have contributed to the abundance of phylotypes (Gammaproteobacteria and Epsilonbactereota), typically associated with the FSB, to become more prevalent within the PV and PS communities.

#### 3.4.3 Distribution of ASVs based on reactor, carbon substrate and response to temperature

Hierarchical clustering analysis was performed to evaluate the distribution of ASVs as a function of reactor geometry and size, carbon substrate, discrete sampling points, and temperature (Figure 7). Five column clusters (C1, C2, C3, C4, and C5) group together based on similarity across different discrete sampling locations from the three reactors at 30 and 10°C. The communities were further grouped into three row clusters (R1, R2, and R3) based on ASVs distribution across samples. Hierarchical cluster analysis showed that at 30°C the PS communities clustered more closely together with the attached CF communities. While at the lower temperature (10°C), the shift in community structure grouped the PS and FSB communities together. This was consistent with the NMDS ordination analysis (Figure 6).

**FIGURE 8:**
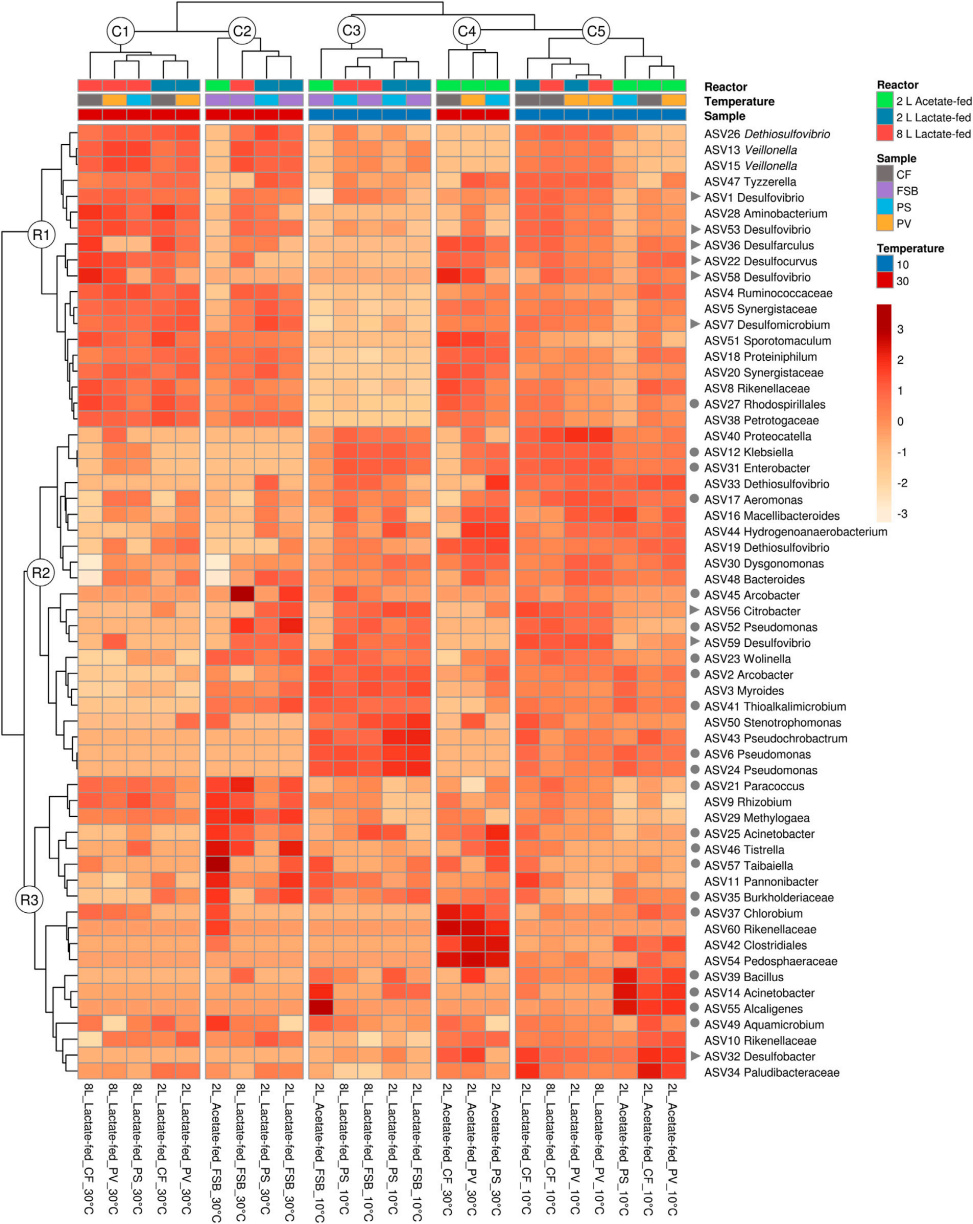
Hierarchical clustering heatmap analysis (distance measured by Euclidean and Ward linkage clustering algorithms), colour intensity reflects the relative abundance of identified OTUs (relative abundance ≥1%), showing the effect of HRT across different microbial communities (CF, PV, PS, and FSB) in the 2 L lactate-fed, 8 L lactate-fed, and 2 L acetate-fed reactors. Putative SRB (▶) and SOB (●) genera are indicated.

A total of 8 putative SRB genera were detected across the three reactors at varying relative abundance. *Desulfovibrio*, *Desulfomicrobium*, *Desulfarculus*, *Desulfocurvus* were the most abundant putative SRB genera detected in the lactate-fed reactors (Figure 8). SRB phylotypes represented within the acetate-fed reactor comprised of *Desulfomicrobium*, *Desulfovibrio*, *Desulfocurvus*, and *Desulfobacter*. As temperature decreased there was a shift in relative abundance of SRB taxa. Interestingly, in the 2 L lactate- and acetate-fed reactors, *Desulfobacter*, a complete-oxidising SRB, increased in relative abundance within the CF and PV communities.

**FIGURE 9:**
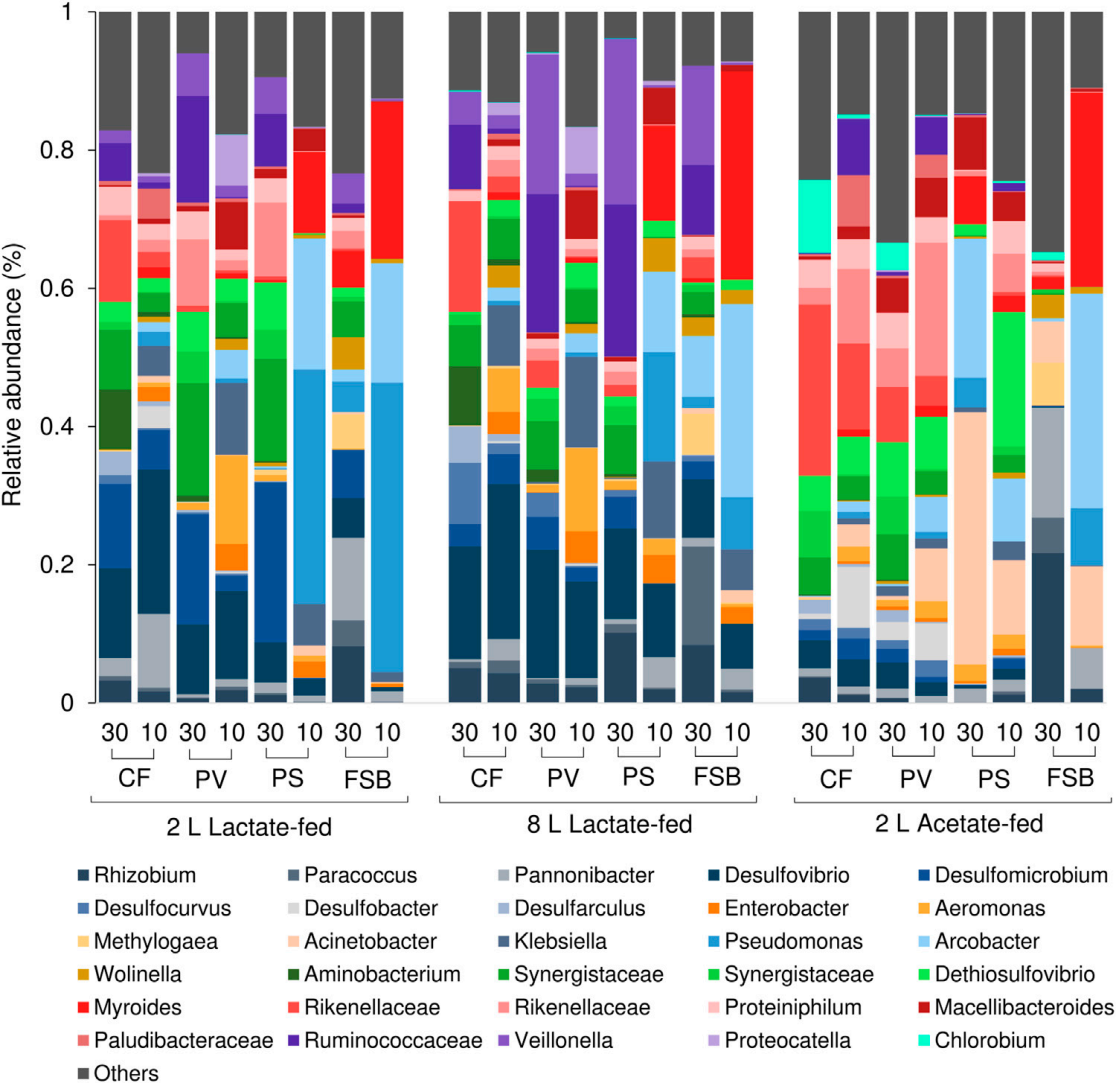
Taxonomic classification at genera level showing the compositional shift in relative abundance of dominant affiliated sequences in the CF, PV, PS, and FSB microbial communities as a function of decreasing temperature from 30 to 10°C in the 2 L lactate-fed, 8 L lactate-fed and 2 L acetate-fed reactors.

The competition between SRB and fermentative microorganisms for lactate has been extensively studied in chemostat experiments (Bertolino et al., 2012; Oyekola et al., 2012). A previous study that evaluated the effect of HRT on process performance and microbial community dynamics revealed that SRB activity was favoured at long residence times while fermentative microorganisms were favoured at short residence times (Marais et al., TBA). In the current study temperature affected both SRB and fermentative populations. At 30°C the CF, PV, and PS communities within the lactate-fed reactors comprised of several obligate anaerobic fermentative microorganisms that included *Veilonella*, *Ruminococcaceae*, and *Synergistaceae* (Figure 9). The high prevalence of fermentative microorganisms is consistent with the lactate fermentation observed within both lactate-fed reactors. A shift in community structure at 10°C resulted in a decrease in the abundance of dominant fermentative taxa, namely Synergistetes and Firmicutes, with a marked increase in facultative anaerobic microorganisms that were affiliated to Gammaproteobacteria (Figure 9). The largest increase in abundance was for *Klebsiella*, *Enterobacter*, and *Aeromonas* within the CF and PV communities in both 2 and 8 L lactate-fed reactors (Figure 9). The CF and PV communities within the acetate-fed reactor at 10°C were dominated by taxa affiliated to the phylum Bacteroidetes (Figure 7). Similarly, there was a shift in community structure in response to the decrease in temperature with a notable reduction in Synergistes accompanied by an increase in the abundance of Ruminococcaceae, Plaudibacteria, and Rikenellacaea (Figure 9). The high prevalence of fermentative taxa belonging to Bacteroidetes and Synergistetes within the acetate-fed reactor were likely enriched by the fermentable yeast extract present within the synthetic feed.

Despite the low abundance of SRB, only accounting for 11% of the CF and PV community within the 2 L acetate-fed reactor at 30°C, the system was still able to achieve relatively high sulphate conversion of 61% (Supplementary Table S1). However, when exposed to low temperature the sulphate conversion decreased to 5%. The poor representation of SRB phylotypes within the acetate-fed reactor revealed the limitation of acetate as a sole carbon source. This is primarily linked to the slower optimal growth rate of acetate-oxidising SRB (doubling time 10–16 h) compared to lactate-oxidising SRB (doubling time 3–10 h) (Celis et al., 2013). Furthermore, acetate selects for a less diverse SRB population, since only a few known SRB are capable of metabolising acetate. Consequently, *Desulfobacter* was the only known complete-oxidising genus that persisted within the acetate-fed reactor even after exposure to low temperature.

In this study it was anticipated that the attached microbial communities (CF) would be less susceptible to change in temperature. Sessile cells encapsulated in EPS matrix are known to be more resilient to change in environmental conditions than free-living cells. At the genus level there was a decrease in the abundance of *Desulfomicrobium*, *Desulfarculus* and *Desulfocurvus* (Figure 9), while *Desulfobacter* and *Desulfovibrio* increased in response to the decrease in reactor temperature (30–10°C), in the lactate-fed reactors. *Desulfocurvus* and *Desulfarculus* were only prevalent within the CF communities at 30°C in the lactate-fed reactors (Figure 9). This indicated a preferential existence within the carbon microfiber attached biofilm communities. At 10°C, *Desulfocurvus* and *Desulfarculus* were reduced to below 1% relative abundance (Figure 9).

#### 3.4.4 Sulphur-oxidising bacterial response to thermal shift

Biofilms are multicellular, structurally complex microbial communities, typically encased and held together by a self-produced extracellular matrix (López et al., 2010). The formation of a biofilm at the air-liquid interface involves intricate spatiotemporal cellular organisation. Since the FSB was intermittently disrupted and reformed, it was anticipated that dynamic shifts in microbial community composition would occur depending on the prevailing temperature. As a new biofilm develops at the air-liquid interface at reduced temperature, the growth of bacterial phylotypes that are more tolerant to colder temperatures were enhanced. The FSB was distinctly different in microbial community composition between 30 and 10°C. A total of 15 putative SOBs were identified across all microbial communities varying in relative abundance. An important aspect in determining the FSB microbial composition is the community succession over time during different developmental stages of the FSB which was not evaluated within the current study.

The most abundant SOB genera detected within the FSB were *Pseudomanas*, *Arcobacter*, and *Parracoccus* across all three reactors. *Acinetobacter* was confined to the acetate-fed reactor and was particularly dominant within the FSB and PS communities at 10°C. *Chlorobium*, an anaerobic photolithotrophic SOB, was only detected at 30°C within the CF and PV communities in the 2 L acetate-fed reactor. At 30°C the FSB comprised of a diverse microbial community with key taxa including *Rhizobium*, *Parracoccus*, *Pannonibacter*, *Arcobacter*, *Desulfomicrobium*, *Desulfovibrio*, and *Wolinella*. Species belonging to the genera *Arcobacter* (Yang et al., 2021), *Acinetobacter* (Potivichayanon et al., 2006), *Parracocus* (Vikromvarasiri et al., 2015), and *Pseudomonas* (Hou et al., 2018) have all been shown to be highly efficient in sulphide oxidation and have been applied in the treatment of sulphide-rich streams.

Similar to the PS zone, there was a clear decline in anaerobic phylotypes, such as *Desulfomicrobium*, *Desulfovibrio*, Synergistetes, and Firmicutes after exposure to low temperatures. At 10°C, *Pseudomonas*, *Myroides*, and *Arcobacter* became highly dominant within the FSB, accounting for approximately 71 ± 9% (mean ± SD) of the total population across all three reactors. *Myroides*, which made up approximately 27 ± 3.9% (mean ± SD) of the population, is recognized as a prolific biofilm producer and has been shown to form a pellicle at the air-liquid interface. A previous study on a *Myroides* strain investigated its ability to facilitate the attachment of *Listeria*. spp to surfaces (Jacobs and Chenia, 2009). It was discovered to have strong hydrophobicity, cell aggregation and coaggregation properties that allowed it to colonise surfaces and form biofilms (Jacobs and Chenia, 2009; Lorenzin et al., 2018; Pompilio et al., 2018). Other taxa such as *Panonibacter*, *Arcobacter*, *Pseudomonas*, and *Paracoccus*, known as biofilm forming denitrifying bacteria, have been used in bioaugmentation of carrier matrices for biological denitrification processes (Grubba and Majtacz, 2020). Both *Pseudomonas* and *Arcobacter* are characterised as nitrate-reducing sulphide-oxidising bacteria (NR-SOB) and are capable of effective sulphide removal (Grubba and Majtacz, 2020). Denitrification coupled to sulphur oxidation plays an important role in the cycling of carbon, nitrogen and sulphur. Since the discovery of the Anammox and Sulfammox (i.e., sulphate reducing ammonium oxidation) processes, the interrelationship between nitrogen and sulphur metabolic reactions within microbial communities are far more complex than was previously thought (Grubba and Majtacz, 2020). While nitrogen speciation was not monitored in this study, the presence of dominant phylotypes associated with denitrification strongly suggests that these processes have been occur within the systems.

Other key genera that were detected within the FSB were *Rhizobium* and *Pannonibacter* which were previously found to be highly abundant within the FSB when the system was operated at a long HRT at 30°C (Marais et al., under review). Although these microorganisms have not been previously associated with sulphur oxidation, they have been implicated in nitrogen fixation and nitrate reduction and may be important in the development and functioning of the FSB.

*Pseudomonas* is highly adaptive to a wide range of environmental conditions and is known to thrive under cold temperatures. Previous studies have reported an increase in biofilm production by *Pseudomonas* when cultivated at 10°C compared to 25°C. Biofilm formation is a common bacterial coping strategy when exposed to stress inducing temperature below optimal range (Vásquez-Ponce et al., 2017).

From an operating perspective, the impact of thermal stress could be mitigated by increasing HRT. This would facilitate longer biomass retention times and ensure that higher sulphide concentrations are maintained within the anoxic bulk volume. Cold acclimatisation over an extended period of continuous operation could also enrich for cold tolerant SRB and SOB consortia. Therefore, diurnal and seasonal fluctuations in temperature while regulating the HRT in favour of sulphate reduction could provide selective pressure to ensure a well-adapted and resilient culture.

The microbial communities that drive the biochemical reactions within these systems are susceptible to thermal stress. Although a considerable reduction in BSR performance occurred when temperature decreased below the critical temperature (15°C), the systems still maintained some BSR activity and selected for cold resilient SRB taxa. After restoring optimal operating conditions at 30°C and longer HRT, all three reactors were able to recover a high sulphate conversion efficiency. The acetate-fed reactor achieved a sulphate conversion of 72%, which was higher than previously reported when operated under similar conditions (Marais et al., 2020c). The increased abundance of *Desulfobacter* during operation at 10°C may have been advantageous for increasing BSR activity. Cold tolerance exhibited by *Desulfobacter* may have facilitated its increase in relative abundance within the system and to compete with microorganisms that dominated at higher temperatures. The continuous fluctuation in diurnal and particularly seasonal temperature that will be experienced at large-scale operations is likely to stimulate more dynamic shifts and development of robust microbial communities in the hybrid LFCR. However, extreme thermal stress below the critical temperature can have detrimental impact on the SRB and SOB microbial communities and overall process performance.

## 4 Conclusion

This study highlights the impact of operating temperature on the overall activity of sulphate reducing and sulphide oxidising processes within the hybrid LFCR. As temperature was incrementally decreased from 30 to 10°C, VSRR and sulphate conversion efficiency decreased. Particularly, on the decrease from 15 to 10°C, thermal stress was observed across all systems through the increase in the activation energy determined through Arrhenius analysis; T_crit_ was observed to lie in this range. Based on these findings, at lower temperatures the system may require operation at a longer residence time in order to compensate for the decline in performance. Most mines in South Africa are in summer rainfall areas so passive flows are likely to be reduced during the colder seasons.

The lactate-fed reactors exhibited a greater resilience to colder temperatures which was reflected through the selection of a dominant diverse SRB community. The reactor performance and ecology data across the 2 and 8 L lactate-fed reactors were consistent and exhibited a similar response to change in temperature. These findings support previous work which demonstrated that aspect ratio and reactor volume had minimal influence on overall process performance and microbial community dynamics. The reduction in performance at low temperature was more pronounced within the acetate-fed system. At 20°C, the decrease in BSR and poor biofilm formation affected the stability and robustness of the system. However, the increased frequency of biofilm disruption (premature disruption), due to the poor formation of a structurally sound FSB, resulted in a higher accumulation of biofilm onto the settling screen and overall recovery within the system. This highlights the importance of effective FSB management through periodically harvesting to facilitate optimal sulphur recovery.

In the hybrid LFCR, the prevailing operating temperature exerts strong environmental selection on microbial community composition. At cold temperatures, overall microbial activity of both SRB and fermentative microorganisms were affected. Differences in the thermal sensitivities between lactate- and acetate-fed reactors led to variation in temperature dependence on VSRR which correlated with Ea values associated with SRB communities’ response to temperature. The FSB comprised of a diverse SOB population that exhibits temporal community shift through different stages of development. At cold temperatures the FSB community was distinctly different favouring the growth and selection of phylotypes more physiologically adapted to cold temperatures. This study demonstrates the importance of temperature as a key variable in the physiological selection and ecological differentiation of SRB and SOB communities within the hybrid LFCR. The data indicates that reduction in performance at low temperature is a combination of reduced metabolic activity and changes in community structure. However, the results did suggest that periods of low temperature could result in a more robust and efficient community as temperatures transition from low to high temperatures, which could have positive implications for real-world applications.

## Data Availability

The datasets presented in this study can be found in online repositories. The names of the repository/repositories and accession number(s) can be found below: https://figshare.com/, 10.25375/uct.19400801; https://www.ncbi.nlm.nih.gov/, PRJNA818253.
