# Supplementary material for: Effect of temperature change on the performance of the hybrid linear flow channel reactor and its implications on sulphate-reducing and sulphide-oxidising microbial community dynamics
Source: Front Bioeng Biotechnol. 2022 Aug 26;10:908463. doi: 10.3389/fbioe.2022.908463 (PMC9458953; doi:10.3389/fbioe.2022.908463)
Supplement: Supplementary file 1 [file Table1.DOCX]

**Supplementary Table 1.** Summary of the overall reactor performance as a function of temperature. The results highlight the biological sulphate reduction and sulphide oxidation kinetics showing the volumetric rates and corresponding conversion.

| Reactor | Temperature (°C) | VSRR  (mmol/L.h) | Sulphate conversion  (%) | VSOR  (mmol/L.h) | | Sulphide conversion  (%) ^c^ | Sulphur Recovery  (%) ^d^ | | Gap-S (%) ^e^ |
| --- | --- | --- | --- | --- | --- | --- | --- | --- | --- |
|  |  |  |  | Maximum^a^ | Average^b^ |  |  |  | |
| 2 L lactate-fed |  | | | | | | | | |
|  | 30 | 0.144 | 66 | 0.077 | 0.033±0.03 | 33 | 30 | | 70 |
|  | 25 | 0.125 | 58 | 0.101 | 0.062±0.03 | 51 | 21 | | 79 |
|  | 20 | 0.116 | 53 | 0.081 | 0.045±0.02 | 44 | 30 | | 70 |
|  | 15 | 0.095 | 44 | 0.069 | 0.041±0.02 | 45 | 32 | | 68 |
|  | 10 | 0.059 | 27 | 0.034 | 0.018±0.01 | 33 | 32 | | 68 |
| 8 L lactate-fed |  | | | | | | | | |
|  | 30 | 0.128 | 61 | 0.108 | 0.038±0.03 | 31 | 30 | | 70 |
|  | 25 | 0.095 | 46 | 0.064 | 0.033±0.02 | 38 | 33 | | 67 |
|  | 20 | 0.080 | 38 | 0.058 | 0.034±0.01 | 38 | 32 | | 68 |
|  | 15 | 0.067 | 32 | 0.043 | 0.029±0.01 | 38 | 27 | | 73 |
|  | 10 | 0.042 | 20 | 0.042 | 0.020±0.01 | 39 | 43 | | 57 |
| 2 L acetate-fed |  | | | | | | | | |
|  | 30 | 0.127 | 61 | 0.081 | 0.034±0.03 | 41 | 29 | | 71 |
|  | 25 | 0.113 | 54 | 0.124 | 0.044±0.03 | 43 | 69 | | 31 |
|  | 20 | 0.070 | 34 | 0.059 | 0.025±0.02 | 33 | >100 | | - |
|  | 15 | 0.047 | 23 | 0.075 | 0.011±0.02 | 21 | - | | - |
|  | 10 | 0.010 | 5 | 0.046 | 0.009±0.02 | 23 | - | | - |
| ^a^ Maximum VSOR measured in the final effluent  ^b^ Average VSOR recorded in the final effluent over the duration of the experimental run (mean±SD)  ^c^ Cumulative sulphide conversion based on the expected sulphide and final effluent  ^d^ Sulphur recovery from the FSB based on sulphide conversion  ^e^ Colloidal sulphur fraction and FSB fragments released into the effluent | | | | | | | | | |
