# Supplementary figures and images for: Effect of temperature change on the performance of the hybrid linear flow channel reactor and its implications on sulphate-reducing and sulphide-oxidising microbial community dynamics
Source: Front Bioeng Biotechnol. 2022 Aug 26;10:908463. doi: 10.3389/fbioe.2022.908463 (PMC9458953; doi:10.3389/fbioe.2022.908463)

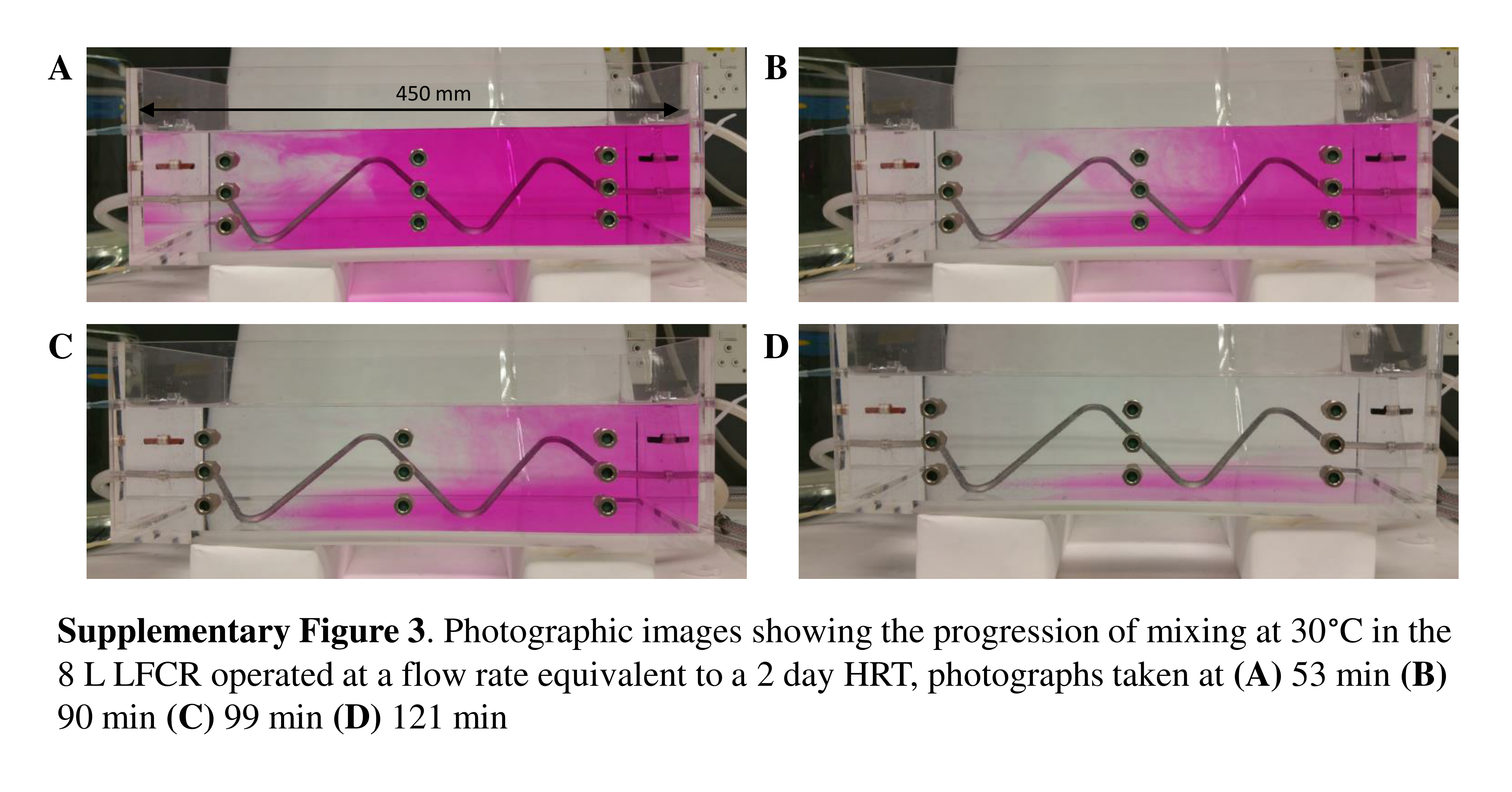

Supplement: Supplementary file 2 [file Image3.TIF]

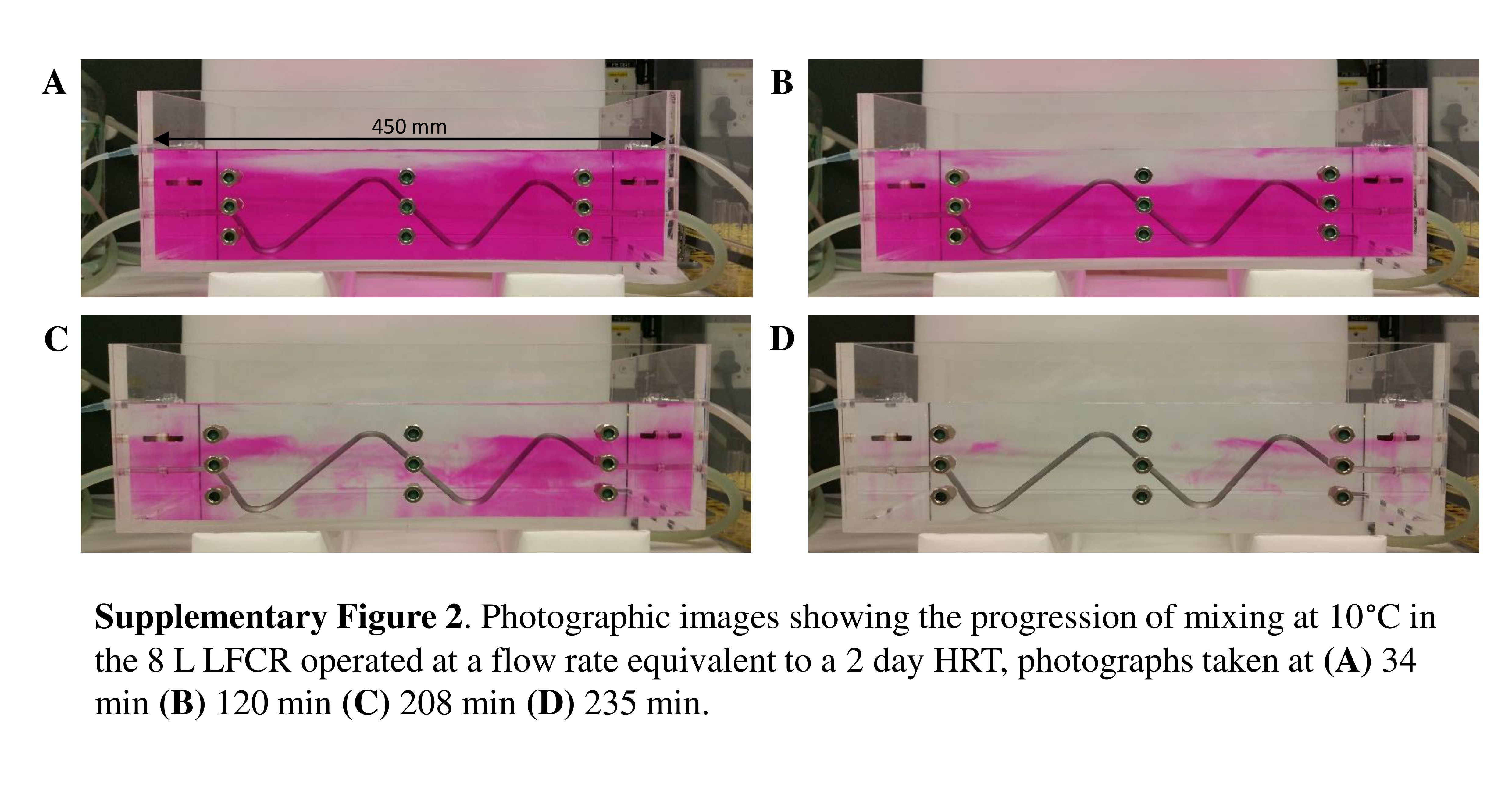

Supplement: Supplementary file 4 [file Image2.TIF]

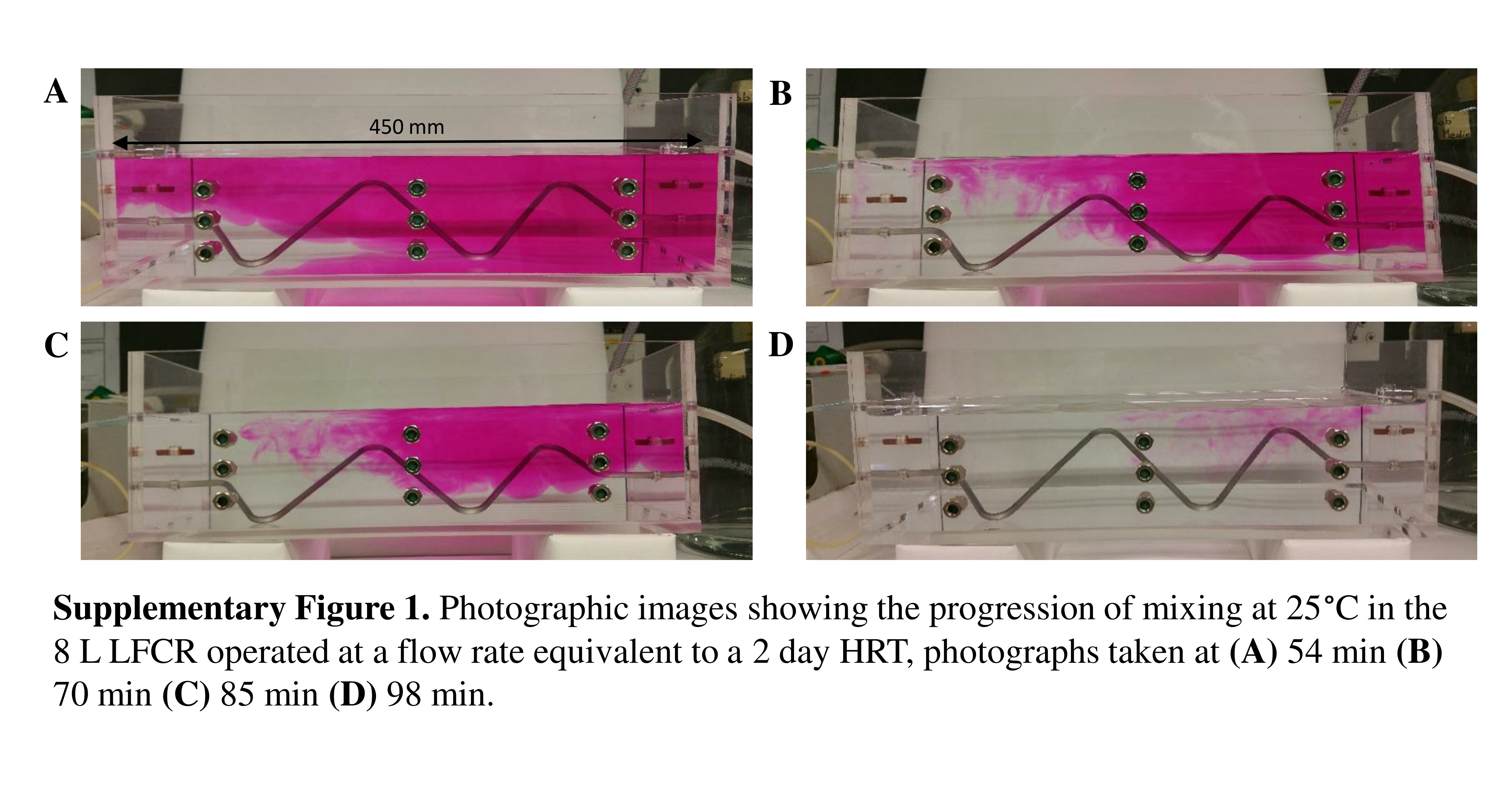

Supplement: Supplementary file 5 [file Image1.TIF]
