# Supplementary material for: Effect of temperature change on the performance of the hybrid linear flow channel reactor and its implications on sulphate-reducing and sulphide-oxidising microbial community dynamics
Source: Front Bioeng Biotechnol. 2022 Aug 26;10:908463. doi: 10.3389/fbioe.2022.908463 (PMC9458953; doi:10.3389/fbioe.2022.908463)
Supplement: Supplementary file 6 [file Table2.DOCX]

**Supplementary Table 2.** Diversity statistics of the microbial communities sampled from the attached biofilm on the carbon microfibers (CF), anoxic planktonic phase (PV), planktonic phase just below at the air-liquid interface (PS) and the floating sulphur biofilm (FSB).

| Reactor | Temperature (°C) | Sample ID | Observed | Shannon | Simpson |
| --- | --- | --- | --- | --- | --- |
| 2 L Lactate-fed |  |  |  |  |  |
|  | 30 | CF | 78 | 3.26 | 0.94 |
|  |  | PV | 60 | 2.73 | 0.90 |
|  |  | PS | 74 | 2.87 | 0.90 |
|  |  | FSB | 77 | 3.43 | 0.95 |
|  | 10 | CF | 169 | 3.78 | 0.95 |
|  |  | PV | 144 | 3.49 | 0.94 |
|  |  | PS | 111 | 2.71 | 0.87 |
|  |  | FSB | 85 | 2.13 | 0.80 |
| 8 L Lactate-fed |  |  |  |  |  |
|  | 30 | CF | 58 | 3.00 | 0.93 |
|  |  | PV | 67 | 2.79 | 0.89 |
|  |  | PS | 70 | 2.63 | 0.90 |
|  |  | FSB | 71 | 3.17 | 0.94 |
|  | 10 | CF | 171 | 3.60 | 0.94 |
|  |  | PV | 140 | 3.43 | 0.94 |
|  |  | PS | 108 | 3.01 | 0.92 |
|  |  | FSB | 87 | 2.46 | 0.83 |
| 2 L Acetate-fed |  |  |  |  |  |
|  | 30 | CF | 73 | 3.16 | 0.92 |
|  |  | PV | 88 | 3.62 | 0.96 |
|  |  | PS | 91 | 3.39 | 0.95 |
|  |  | FSB | 65 | 3.11 | 0.92 |
|  | 10 | CF | 166 | 3.49 | 0.95 |
|  |  | PV | 156 | 3.43 | 0.81 |
|  |  | PS | 116 | 2.37 | 0.94 |
|  |  | FSB | 83 | 2.31 | 0.83 |
